# Atypical flavobacteria recovered from diseased fish in the Western United States

**DOI:** 10.3389/fcimb.2023.1149032

**Published:** 2023-04-19

**Authors:** Taylor I. Heckman, Zeinab Yazdi, Eric K. Pomaranski, Fernanda de Alexandre Sebastião, Kaveramma Mukkatira, Brent M. Vuglar, Kenneth D. Cain, Thomas P. Loch, Esteban Soto

**Affiliations:** ^1^ Department of Medicine and Epidemiology, School of Veterinary Medicine, University of California, Davis, Davis, CA, United States; ^2^ Fisheries, Embrapa Amazônia Ocidental, Manaus, Amazonas, Brazil; ^3^ Fish Health Laboratory, California Department of Fish and Wildlife, Rancho Cordova, CA, United States; ^4^ Department of Fish and Wildlife Sciences, College of Natural Resources, University of Idaho, Moscow, ID, United States; ^5^ Department of Fisheries and Wildlife, College of Agriculture and Natural Resources, Department of Pathobiology and Diagnostic Investigation, College of Veterinary Medicine, Michigan State University, East Lansing, MI, United States

**Keywords:** Flavobacteriales, Flavobacterium, Chryseobacterium, 16S rRNA, *gyrB*, flavobacteria

## Abstract

Flavobacterial diseases, caused by bacteria in the order Flavobacteriales, are responsible for devastating losses in farmed and wild fish populations worldwide. The genera *Flavobacterium* (Family *Flavobacteriaceae*) and *Chryseobacterium* (*Weeksellaceae*) encompass the most well-known agents of fish disease in the order, but the full extent of piscine-pathogenic species within these diverse groups is unresolved, and likely underappreciated. To identify emerging agents of flavobacterial disease in US aquaculture, 183 presumptive *Flavobacterium* and *Chryseobacterium* isolates were collected from clinically affected fish representing 19 host types, from across six western states. Isolates were characterized by 16S rRNA gene sequencing and phylogenetic analysis using the *gyrB* gene. Antimicrobial susceptibility profiles were compared between representatives from each major phylogenetic clade. Of the isolates, 52 were identified as *Chryseobacterium* species and 131 as *Flavobacterium*. The majority of *Chryseobacterium* isolates fell into six clades (A-F) consisting of ≥ 5 fish isolates with ≥ 70% bootstrap support, and *Flavobacterium* into nine (A-I). Phylogenetic clades showed distinct patterns in antimicrobial susceptibility. Two *Chryseobacterium* clades (F & G), and four *Flavobacterium* clades (B, G-I) had comparably high minimal inhibitory concentrations (MICs) for 11/18 antimicrobials tested. Multiple clades in both genera exhibited MICs surpassing the established *F. psychrophilum* breakpoints for oxytetracycline and florfenicol, indicating potential resistance to two of the three antimicrobials approved for use in finfish aquaculture. Further work to investigate the virulence and antigenic diversity of these genetic groups will improve our understanding of flavobacterial disease, with applications for treatment and vaccination strategies.

## 1 Introduction

The order Flavobacteriales comprises a rapidly expanding group of gram-negative bacteria. Many species within the order are environmental bacteria and populate a wide range of aquatic and terrestrial habitats worldwide (Bernardet, 2015). However, some genera contain established or emerging pathogens of animals and humans, such as *Flavobacterium, Chryseobacterium, Tenacibaculum, Elizabethkingia*, and *Ornithobacterium* (Loch and Faisal, 2015a)*. Flavobacterium* and *Chryseobacterium* hold particular significance for aquaculture and fish health, as they include bacterial species responsible for devastating losses in farmed and wild fish populations, with consequently substantial economic and ecological effects. Acute outbreaks of piscine flavobacteriosis can cause mortality rates upwards of 70%, while subacute or chronic cases result in persistent low-level mortalities (Austin and Austin, 2016). Flavobacteria may also cause lingering deficits in surviving fish, or act as spoilage organisms to further reduce fish fitness and production outputs (Kent et al., 1989; Madsen et al., 2001; Bernardet et al., 2005). Flavobacterial fish disease was first reported in 1922 (Davis, 1922), and in the hundred years since, there has been extensive scientific effort towards preventing and controlling outbreaks. These efforts, however, have been compromised by the rapid expansion and continual restructuring of the genera, species, and strains associated with the disease.

The genus *Chryseobacterium* was first proposed to accommodate six species excluded from *Flavobacterium* in 1994 (Vandamme et al., 1994). In 2006, following further revision of the genera, *Chryseobacterium* included 10 species (Bernardet et al., 2006), and *Flavobacterium*, 26 (Bernardet and Bowman, 2006). Both genera have seen extensive additions over the last decade, and at the time of writing, the number of validly published, nonsynonymous names has soared to 136 *Chryseobacterium* species and 269 *Flavobacterium* species (Parte et al., 2020). The expansion in newly described species has been matched by increasing reports of new agents associated with fish and fish disease. The most well-established flavobacterial piscine pathogens are *Flavobacterium psychrophilum*, the causative agent of bacterial coldwater disease, *F. branchiophilum* the agent of bacterial gill disease, and *F. columnare*, of columnaris disease (Loch and Faisal, 2015a; Wahli and Madsen, 2018). *Flavobacterium columnare* has been recently reclassified into four related species: *F. columnare, F. covae, F. davisii* and *F. oreochromis* (LaFrentz et al., 2022). In addition to these historical pathogens, however, almost fifty other species of *Flavobacterium* and *Chryseobacterium* have been implicated in fish disease (**Tables 1**, **2**). For example, *Flavobacterium tructae, F. oncorhynchi, F. plurextorum, Chryseobacterium piscicola, C. scopthalmum, C. chaponense*, and *C. indologenes* have been isolated from a diverse range of both healthy and diseased, cultured or wild fish worldwide (Loch and Faisal, 2015a; Sebastião et al., 2019a). *Chryseobacterium* species such as *C. indologenes* may also be capable of causing disease in other animals, including humans (Vaneechoutte et al., 2007; Holmes et al., 2013; Lin et al., 2019). Some of these atypical flavobacterial strains have been assessed for their pathogenic potential through laboratory-controlled challenges in fish hosts (**Tables 1**, **2**). However, the majority are linked to disease by nature of their isolation from the external or internal tissues of clinically affected fish. Infected fish often display signs mimicking those caused by established etiologic agents, including skin ulceration, fin erosion, and gill necrosis (Loch and Faisal, 2016b; Jung et al., 2022; Malick et al., 2022). Additionally, fish may be concurrently infected by more than one species, and atypical flavobacteria may not be easily distinguished by conventional diagnostic methods (Bernardet et al., 2005; Bruce et al., 2020). Current limitations in epidemiological and pathological data obfuscate the risk these potential pathogens pose to aquaculture.

**Table 1:** *Flavobacterium* species associated with fish reported in the literature to date.

	Species	Hosts reported	References
Experimentally demonstrated virulence in fish	*F. johnsonieae*	Barramundi (*Lates calcarifer*) Koi (*Cyprinus rubrofuscus*) Longfin eels (*Anguilla mossambica*) Russian sturgeon (*Ascipenser. gueldenstaedtii*) Salmonids	(Christensen, 1977; Carson et al., 1993; Soltani et al., 1994; Rintamäki-Kinnunen et al., 1997; Flemming et al., 2007)
	*F. tructae* (syn. *F. spartansii)*	Rainbow trout (*Oncorhynchus mykiss*) Steelhead (*Oncorhynchus mykiss*) Chinook salmon (*Oncorhynchus tshawytscha*) Lake trout (*Salvelinus namaycush*)	(Loch and Faisal, 2014c, Loch and Faisal, 2014a; Zamora et al., 2014; Loch and Faisal, 2016b; Kämpfer et al., 2020)
Disease-associated	*F. araucananum*	Atlantic salmon (*Salmo salar*)	(Kämpfer et al., 2012)
	*F. bernardetii*	Rainbow trout (*O. mykiss*)	(Saticioglu et al., 2021b)
	*F. bizetiae*	Diseased fish	(Mühle et al., 2020)
	*F. chilense*	Rainbow trout (*O. mykiss*)	(Kämpfer et al., 2012)
	*F. frigidimaris*	Mottled sculpin (*Cottus bairdii*) Brown trout (*Salmo trutta*) Lake trout (*S. namaycush*) Chinook salmon eggs and milt (*O. tshawytscha*)	(Loch et al., 2013; Loch and Faisal, 2014a, Loch and Faisal, 2018)
	*F. hydatis*	Russian sturgeon (*Ascipenser gueldenstaedtii*) Salmonids	(Strohl and Tait, 1978; Bernardet and Bowman, 2006; Mantareva et al., 2022)
	*F. inkyongense*	Chocolate cichlid (*Hypselecara temporalis*)	(Sebastião et al., 2019a)
	*F. kayseriense*	Rainbow trout (*O. mykiss*)	(Saticioglu et al., 2021a)
	*F. oncorhynchi*	Rainbow trout (*O. mykiss*) Chinook salmon (*O. tshawytscha*) Brown trout (*S. trutta*) Brook trout (*Salmo fontinalis*) Mottled sculpin (*Cottus bairdii*) Lake whitefish (*Coregonus clupeaformis*) Sea lamprey (*Petromyzon marinus*) Human (*Homo sapiens*),	(Zamora et al., 2012a; Loch et al., 2013; Loch and Faisal, 2018; Ferreira et al., 2022)
	*F. piscis*	Rainbow trout (*O. mykiss*) Chinook salmon (*O. tshawytscha*)	(Zamora et al., 2014; Loch and Faisal, 2016c, Loch and Faisal, 2018)
	*F. plurextorum*	Rainbow trout (*O. mykiss*) Steelhead (*O. mykiss*), Brown trout (*S. trutta*) Brook trout (*Salmo fontinalis*) Chinook salmon (*O. tshawytscha*), Chum salmon eggs (*Onchorynchus keta*) Sea lamprey (*Petromyzon marinus*)	(Zamora et al., 2013a; Loch and Faisal, 2014a)
	*F. succinicans*	Rainbow trout (*O. mykiss*) Chinook salmon (*O. tshawytscha*) Arctic char (*Salvelinus alpinus*) Atlantic salmon (*S. salar*) Carp (*C. carpio*)	(Anderson and Ordal, 1961; Good et al., 2015; Adamek et al., 2018; Loch and Faisal, 2018; Einarsdottir et al., 2020)
	*F. turcicum*	Rainbow trout (*O. mykiss*)	(Saticioglu et al., 2021a)
Fish-associated	*F. aquidurense*	Chinook (*O. tshawytscha*) hatchery stack water	(Loch and Faisal, 2018)
	*F. aquatile*	Nile tilapia (*Oreochromis niloticus*)	(Mwega et al., 2019)
	*F. aquaticum*	Catla (*Catla catla*)	(Verma and Rathore, 2015)
	*F. branchiarum*	Rainbow trout (*O. mykiss*)	(Zamora et al., 2013b)
	*F. branchiicola*	Rainbow trout (*O. mykiss*)	(Zamora et al., 2013b)
	*F. collinsii*	Rainbow trout (*O. mykiss*)	(Zamora et al., 2013b)
	*F. erciyesense*	Rainbow trout (*O. mykiss*)	(Saticioglu, 2021)
	*F. hibernum*	Chinook salmon eggs (*O. tshawytscha*)	(Loch and Faisal, 2018)
	*F. indicum*	Nile tilapia (*O. niloticus*)	(Mwega et al., 2019)
	*F. ichthyis*	Fishpond	(Chen et al., 2020)
	*F. limicola*	Chinook salmon (*O. tshawytscha*) hatchery stack water	(Loch and Faisal, 2018)
	*F. muglaense*	Rainbow trout (*O. mykiss*)	(Duman et al., 2021)
	*F. oryzae*	Nile tilapia (*O. niloticus*)	(Mwega et al., 2019)
	*F. psychrolimnae*	Walleye (*Sander vitreus)* Chinook (*O. tshawytscha*) hatchery stack water	(Loch et al., 2013; Loch and Faisal, 2018)
	*F. salmonis*	Atlantic salmon (*S. salar*) fry culture water	(Kämpfer et al., 2020)
	*F. saccharophilum*	Chinook salmon (*O. tshawytscha*) eggs and fry	(Loch and Faisal, 2018)
	*F. suncheonense*	Carp (*C. carpio*) Catla (*Catla catla*) Rohu (*Labeo rohita*)	(Verma and Rathore, 2015)
	*F. tilapiae*	Tilapiine cichlid fish culture pond	(Chen et al., 2013)

Species included have at least one published record of an isolate identified from fish or fish habitats by whole genome analysis, or by 16S rRNA gene sequencing with ≥ 99% sequence identity to the given species. Species with experimentally demonstrated virulence include at least one strain tested for ability to cause disease in fish through laboratory challenges that resulted in lesions, clinical signs, or mortality.

**Table 2:** *Chryseobacterium* species associated with fish reported in the literature to date.

	Species	Hosts reported	References
Experimentally demonstrated virulence in fish	*C. aahli*	Brook trout (*Salvelinus fontinalis*) Lake trout (*Salvelinus namaycush*) Chinook salmon eggs (*Oncorhynchus tshawytscha*)	(Loch, 2012; Loch and Faisal, 2014b, Loch and Faisal, 2015b, Loch and Faisal, 2018)
	*C. balustinum*	Halibut (*Hippoglossus hippoglossus*) Dace (*Leuciscus leuciscus*)	(Harrison, 1929; Brisou, 1959)
	*C. chaponense*	Atlantic salmon (*Salmo salar*) Rainbow trout (*Oncorhynchus mykiss*) Chinook salmon (*O. tshawytscha*)	(Kämpfer et al., 2011; Loch and Faisal, 2015b)
	*C. cucumeris*	Pond loach (*Misgurnus anguillicaudatus*)	(Kim et al., 2020)
	*C. gleum*	Rohu *(Labeo rohita)* Catla (*Catla catla*) Nile tilapia (*Oreochromis niloticus*) Humans (*Homo sapiens*)	(Hugo et al., 2019; Malick et al., 2022)
	*C. indologenes*	Yellow perch (*Perca flavescens*) Nile tilapia (*O. niloticus*) Chinook salmon (*O. tshawytscha*) Humans (*H. sapiens*) Douc langur (*Pygathrix nemaeus*) Ball Python (*Python regius*) Soft-shell turtle (*Trionyx sinensis*) Mute Swan (*Cygnus olor*) Ring-Billed Gull (*Larus delawarensis*) Soft ticks (*Ornithodoros moubata*) Marine invertebrates	(Burešová et al., 2006; Maravić et al., 2013; Pridgeon et al., 2013; YiBin et al., 2018; Hugo et al., 2019; Mwega et al., 2019; Sebastião et al., 2019b; Malka et al., 2020; Tamai et al., 2021; Chong et al., 2022)
	*C. joostei*	Atlantic salmon (*S. salar*) Stellate sturgeon (*Acipenser stellatus*) Nile tilapia (*O. niloticus*)	(Bernardet et al., 2005; Gholamhosseini et al., 2018; Mwega et al., 2019)
	*C. oncorhynchi*	Siberian sturgeon (*Acipenser baerii)* Rainbow trout (*O. mykiss*)	(Zamora et al., 2012a; SuXu et al., 2019)
	*C. piscicola*	Rainbow trout (*O. mykiss*) Atlantic salmon (*S. salar*) Brown trout (*Salmo trutta*)	(Ilardi et al., 2009, Ilardi et al., 2010; Loch et al., 2013; Loch and Faisal, 2015b)
	*C. piscium*	Chinook salmon (*O. tshawystcha*) Cisco (*Coregonus artedi*) Steelhead (*O. mykiss*) Lake sturgeon eggs (*Acipenser fulvescens*) Wild marine fish	(de Beer et al., 2006; Chalupnicki et al., 2015; Loch and Faisal, 2015b, Loch and Faisal, 2016c)
	*C. scopthalmum*	Turbot (*Scopthalmus maximus*) Black rockcod (*Notothenia coriiceps*) Chinook salmon (*O. tshawytscha*) Rainbow trout (*O. mykiss*) Golden mahseer (*Tor putitora*)	(Mudarris and Austin, 1989; Mudarris et al., 1994; Bernardet et al., 2006; Avendaño-Herrera et al., 2016; Loch and Faisal, 2016c; Shahi et al., 2018)
	*C. viscerum*	Rainbow trout (*O. mykiss*) Muskellunge (*Esox masquinongy*) Mottled sculpin (*Cottus bairdii*)	(Zamora et al., 2012d; Loch et al., 2013; Loch and Faisal, 2015b)
Disease-associated	*C. aquaticum*	Rainbow trout (*O. mykiss*) Brown trout (*Salmo trutta*) Siberian sturgeon (*Acipenser baerii*) Green sturgeon (*A. medirostris*)	(Bernardet et al., 2005; Loveland-Curtze et al., 2010; Hugo et al., 2019; Sebastião et al., 2019b; Saticioglu et al., 2021c)
	*C. bernardetii*	Rainbow trout (*O.* mykiss) Humans (*H. sapiens*)	(Holmes et al., 2013; Saticioglu et al., 2021b)
	*C. indoltheticum*	Steelhead (*O. mykiss*) Coho salmon (*Oncorhynchus kisutch*) Cisco (*C. artedi*) Chinook salmon eggs (*O. tshawytscha*)	(Loch et al., 2013; Loch and Faisal, 2018)
	*C. shigense*	Rainbow trout (*O. mykiss*)	(Zamora et al., 2012b; Loch et al., 2013)
	*C. siluri*	Eastern catfish (*Silurus asotus*)	(Oh et al., 2020)
	*C. tructae*	Rainbow trout (*O. mykiss*)	(Zamora et al., 2012c)
	*C. yeoncheonense*	Brown trout (*S. trutta*) Chinook salmon eggs (*O. tshawytscha*)	(Loch and Faisal, 2015b, Loch and Faisal, 2018)
Fish-associated	*C. vrystaatense*	Mottled sculpin (*Cottus bairdii*) Sea lamprey (*Petromyzon marinus*)	(Loch et al., 2013)
	*C. hominis* (syn. *C. arothri*)	White-spotted puffer (*Arothron hispidus*) Humans (*H. sapiens*)	(Vaneechoutte et al., 2007; Campbell et al., 2008; Kämpfer et al., 2009)
	*C. haifense*	Lake sturgeon eggs (*A. fulvescens*)	(Chalupnicki et al., 2015)
	*C. limigenitum*	Chinook salmon eggs (*O. tshawytscha*)	(Loch and Faisal, 2018)

Species included have at least one published record of an isolate identified from fish or fish habitats by whole genome analysis, or by 16S rRNA gene sequencing with ≥99% sequence identity to the given species. Species with experimentally demonstrated virulence include at least one strain tested for ability to cause disease in fish through laboratory challenges that resulted in lesions, clinical signs, or mortality.

To address these knowledge gaps, international efforts in surveillance and characterization of fish associated *Flavobacterium* and *Chryseobacterium* strains are gaining in momentum. Methods to identify isolates more conclusively through biochemical or molecular means continue to be improved, and the advent of next generation sequencing has aided the discovery of genetic factors related to virulence, immunogenicity, and antimicrobial resistance, together supporting development of new tools for disease treatment and prevention (García-López et al., 2019; Saticioglu et al., 2021c; Jung et al., 2022; Malick et al., 2022). Antimicrobial resistance is a particularly notorious issue for both human and animal flavobacterial infections, as high intrinsic resistance to multiple classes of antimicrobials is archetypal of some genera (Verner-Jeffreys et al., 2017). The susceptibility profiles of historic pathogens, such as *F. psychrophilum* and *F. columnare* are well-studied, but there is a lack of information on antimicrobial resistance (AMR) characteristics for more recently described species.

In the United States, such newly described *Flavobacterium* and *Chryseobacterium* species have been identified in the Great Lakes region, through several studies investigating flavobacterial community composition in salmonids at different life stages (Loch et al., 2013; Loch and Faisal, 2014a; Loch and Faisal, 2015b; Loch and Faisal, 2016a; Loch and Faisal, 2016c; Loch and Faisal, 2018). Through these studies, species previously described only in other continents were identified, indicating the presence of these flavobacteria in the US. There is a paucity of information, however, on the distribution and prevalence of atypical *Flavobacterium* and *Chryseobacterium* in other regions of the country. The Western United States has a high concentration of salmonid production, an industry perpetually plagued by flavobacterial disease (Wahli and Madsen, 2018; United States Department of Agriculture, 2019). Despite this, only one study has investigated the circulating diversity of novel fish-associated flavobacteria in the region, limited to a sampling of *Chryseobacterium* spp. in California (Sebastião et al., 2019b). To better understand potential emerging threats to aquaculture in Western United States, we aimed to assess the diversity of *Chryseobacterium* and *Flavobacterium* associated with fish disease in the region. Yellow-pigmented bacteria isolated from clinically affected fish in six western states were collected and screened by sequencing of the 16S rRNA gene. Isolates were then typed by *gyrB* analysis to identify major clades associated with diseased fish for further phenotypic and antimicrobial characterization. Sequencing of the ubiquitous 16S rRNA gene remains the most common method for bacterial classification to the genus level but lacks the phylogenetic power to resolve closely related species in genera like *Flavobacterium* and *Chryseobacterium* (Janda and Abbott, 2007; Kumru et al., 2020). The *gyrB* gene typically has a faster evolution rate than the 16S rRNA gene, resulting in higher genetic heterogeneity and improved discrimination at the species level for bacterial genotyping (Wang et al., 2007; Galloway-Peña et al., 2014; Reichley et al., 2017).

## 2 Materials and methods

### 2.1 Bacteria

Over a period of 5 years, diverse yellow pigmented bacterial (YPB) strains were compiled from fish exhibiting signs of flavobacterial disease in the Western United States, either submitted to the Aquatic Animal Health Laboratory at the University of California, Davis, or collected in-house from diagnostic cases. Historical untyped or unusual YPB were also accepted, with initial collection dates ranging from 1991-2022. Bacteria were isolated from external lesions or internal organs and submitted as pure cultures on solid media. Bacteria of interest were considered to be those that exhibited morphologies typical for flavobacteria (yellow to orange pigmentation, gram-negative, catalase and oxidase positive), but were not initially identified as *F. columnare* or *F. psychrophilum* by species-specific qPCR (“atypical”) or other diagnostic methods. Bacterial cultures were expanded in 5 mL of *Flavobacterium columnare* growth media (FCGM; Farmer, 2004), diluted Mueller-Hinton media (dMH; Sigma-Aldrich, St. Louis, MO, USA), Modified Shieh media (MS; LaFrentz and Klesius, 2009) or tryptone yeast extract salts media (TYES; University of California Davis, Biological Media Services, USA) for 24-72h at 20°C with shaking. Bacterial suspensions were saved in 1 mL aliquots with 20% glycerol at −80°C until further use.

### 2.2 Genomic DNA extraction

Bacterial strains were revived from freezer stocks on TYES or MS agar at 20°C for 72-96 h. A single isolated colony was then transferred into 5 mL broth media for expansion at 20°C for 24-72 h with shaking. One milliliter of the expanded bacterial suspension was centrifuged for 10 min at 5,000 *x g* (7500 rpm). Genomic DNA (gDNA) was extracted from the concentrated pellet using the DNeasy^®^ Blood and Tissue kit (Qiagen, Germantown, MD, USA) following manufacturer recommendations for gram-negative bacteria. The quality and quantity of recovered DNA was assessed using a NanoDrop™ One Microvolume UV-Vis Spectrophotometer (Thermo Fisher Scientific™, Waltham, MA USA) and samples with 260/280 ratios of 1.8-2.0 were cryogenically stored (-20°C) until further analysis.

### 2.3 Molecular identification

Primers previously used for identification and typing of unknown *Flavobacteriales* were employed (**Table 3**, Sebastião et al., 2019b). Amplification by PCR was conducted using either Phusion™, Platinum™ *Taq* or DreamTaq™ DNA polymerases (Thermo Fisher Scientific™, Waltham, MA USA) following manufacturer’s instructions. Phusion reaction mixtures (20 µL) consisted of 4 µL 5X Phusion™ HF Buffer, 0.4 µL dNTPs (10 mM), 1 µL each of the forward and reverse primers (10 µM), 0.2 µL Phusion™ High-Fidelity DNA polymerase (2 U/µL), 1-2 µL of template DNA and up to 20 µL diethyl pyrocarbonate (DEPC) water. Thirty seconds of initial denaturation at 98°C were followed by 35 cycles of 98°C for 10 s, 57°C for 30 s, and 72°C for 30s, with a final extension at 72°C for 5 min. For difficult templates, 3% DMSO was included in Phusion reaction mixtures and annealing temperatures adjusted to 6°C below the suggested melting temperature for each primer set (**Table 3**). Platinum *Taq* reaction mixtures (25 µL) consisted of 2.5 µL 10X high fidelity PCR buffer, 1 µL MgSO_4_, 0.5 µL dNTPs (10 mM), 0.5 µL each of the forward and reverse primers (10 µM), 0.1 µL Platinum™ *Taq* DNA polymerase high fidelity (5 U/µL), 1-2 µL of template DNA and up to 25 µL DEPC water. Thirty seconds of initial denaturation at 94°C were followed by 35 cycles of 94°C for 15 s, 51°C for 30 s, and final extension at 68°C for 1 minute. DreamTaq™ reaction mixtures (25 µL) consisted of 12.5 µl 2X DreamTaq Green PCR Master Mix, 1µL of each forward and reverse primer (10 µM), 7 µl of DNA, and 3.5 µl of DEPC water to volume. Five seconds of initial denaturation at 95°C were followed by 35 cycles of 95°C for 30 s, 55°C for 10 s, and 72°C for 60s, with a final extension at 72°C for 5 min.

**Table 3:** Primers used for bacterial identification and typing.

	Primer name	Sequence (5’ – 3’)	Tm (°C) Platinum *Taq™*	Tm (°C) Phusion ™	Amplicon size (bp)	
16S	8F	AGA GTT TGA TYM TGG CTC AG	51	58.2	899	(Lane et al., 1985; Felske et al., 1997; Ben-Dov et al., 2006)
	907R	CCG TCA ATT CMT TTR AGT TT	51	57.1		
*gyrB*	40F	AGT ATT CAG GCA TTA GAA GG	50.5	57.6	1,723	(Sebastião et al., 2019b)
	1763R	TCT CCA AGA CCT TTA TAA CG	51.4	57.5		

Amplification reactions were electrophoresed through 1% agarose gels supplemented with SYBR^®^ Safe DNA gel stain (Invitrogen, Waltham, MA USA; 1 µL/mL) alongside concurrently run molecular weight standards (Quick-Load^®^ Purple 100 bp DNA Ladder, New England BioLabs, Ipswich, MA, USA) and visualized under ultraviolet light to confirm the presence of appropriately sized bands. PCR products were purified using the QIAquick PCR Purification Kit (Qiagen, Germantown MD, USA) and their concentration and purity assessed by Nanodrop. Purified products and corresponding forward and reverse primers were diluted and submitted for Sanger sequencing at the University of California, Davis Sequencing facility (UC Davis, CA, USA) or through GENEWIZ (South San Francisco, CA, USA). Forward and reverse sequences were imported into Geneious Prime (2022.2.2), trimmed to a 0.05 error probability limit, and *de novo* assembled. Isolate sequences for each gene were aligned by MUSCLE using default settings and trimmed to the region of quality bases shared by all isolate sequences. The nucleotide Basic Local Alignment Search Tool in Geneious (blastn; Altschul et al., 1990) was used to compare each isolate’s 16S rRNA gene fragment to a local database populated by 136 *Chryseobacterium* and 269 *Flavobacterium* type strains. The “closest known species” was determined based on the type-strain with the highest pairwise identity (PI) to the isolate along the entire sequenced fragment. Isolates that were not within the *Flavobacterium* or *Chryseobacterium* genera were removed from further analyses.

### 2.4 Phylogenetic analysis

Phylogenetic analyses using the *gyrB* gene were performed to compare evolutionary relationships between isolates and established species, and to identify fish disease associated clusters. Analyses were performed separately for *Flavobacterium* and *Chryseobacterium* isolates. A representative isolate sequence for the *gyrB* gene fragment was used in BLAST searches of a localized database populated by validly published *Flavobacterium* and *Chryseobacterium* species with whole genomes available for download from GenBank. Where type-strain genomes were absent, NCBI reference genomes were used as available. The database represented 178 *Flavobacterium* species and 94 *Chryseobacterium* species isolated from animal hosts or the environment (**Supplementary Table 1**). The resulting sequences from isolates and published strains were exported to MEGA-X (Kumar et al., 2018) and aligned by MUSCLE using default settings. A maximum likelihood tree was generated using the General Time Reversible model (Nei and Kumar, 2000) with a gamma distribution and invariable rates (GTR+G+I), selected based on Bayesian and corrected Akaike Information Criterion in MEGA-X. The percentage bootstrap confidence levels were calculated from 1000 re-samplings of the original data. Major clades were defined as clusters containing ≥ 5 fish isolates with ≥ 70% bootstrap support. Phylogenetic trees were exported from MEGA as Newick files and formatted and annotated in iTOL (Letunic and Bork, 2019).

### 2.5 Phenotypic characterization

A representative isolate from each of the major clades (n=15) was used for preliminary phenotypic comparison by conventional methods. Colony morphology was compared by assessing bacterial growth on TYES after 72 h at 20°C, while hemolytic capability was investigated by growth on trypticase soy agar supplemented with 5% sheep blood (SBA; University of California Davis, Biological Media Services, USA). Gram staining was carried out using established protocols. Catalase activity was assessed by application of 3% H_2_O_2_ and cytochrome oxidase by the swab test using tetramethyl-p-phenylenediamine dihydrochloride reagent (Remel, Lenexa, Kansas, USA).

### 2.6 Antimicrobial susceptibility profiling

Antimicrobial susceptibility profiles were investigated for three representative isolates from each of the major clades of atypical *Flavobacterium* and *Chryseobacterium* (n=39) recovered from clinically infected fish in the Western United States using the broth microdilution method. The minimal inhibitory concentrations for 18 antimicrobials were determined using the Sensititre Avian 1F plates (Thermo Fisher Scientific™, Waltham, MA USA) following CLSI guidelines established for *F. psychrophilum* (CLSI, 2020). Briefly, bacteria were revived from freezer stocks on TYES or MS agar at 20°C for 72 h and used to generate a 0.5 McFarland standard (~1.5 x 10^8^ CFU/mL) in sterile phosphate buffered saline (1X PBS). Bacterial suspensions were diluted 1:1,000 in cation-adjusted diluted Mueller Hinton media (CAT-dMH; BD Biosciences, San Jose, CA, USA) and 50 µL aliquoted into each well of the Sensititre plate. Inoculated plates were incubated at 20°C and read at 96 h. The minimal inhibitory concentration (MIC) was defined as the lowest concentration for which no bacterial growth was grossly observed.

## 3 Results

### 3.1 Bacterial identification

A total of 183 isolates presumptively ascribed to the genera *Flavobacterium* and *Chryseobacterium* were collected for analysis in this study. These isolates were collected over the span of 31 years from 19 fish types in 6 western states – California (CA), Colorado (CO), Washington (WA), Oregon (OR), Utah (UT), and Idaho (ID) (**Supplementary Tables 3, 4**). Of the 183 isolates, 52 were identified as *Chryseobacterium* and 131 as *Flavobacterium* by BLASTN of partial 16S rRNA gene sequences. Most isolates (125/183) shared ≥ 99% pairwise nucleotide identity to a type-strain in the sequenced fragment. However, 44 *Flavobacterium* isolates and 14 *Chryseobacterium* isolates fell below this threshold, with pairwise identities ranging from 95.8-98.9%. The 16S rRNA gene was highly conserved within the genera. Within *Flavobacterium*, the 269 type-strains and 131 isolates (n = 400 total) shared average 92.5% nucleotide identity within the sequenced fragment. Similarly, within the *Chryseobacterium* genus, the 136 type-strains and 52 isolates (n = 188 total) shared a pairwise identity of 94.8%.

### 3.2 Phylogenetic analyses

#### 3.2.1 Flavobacterium

**Figure 1:**
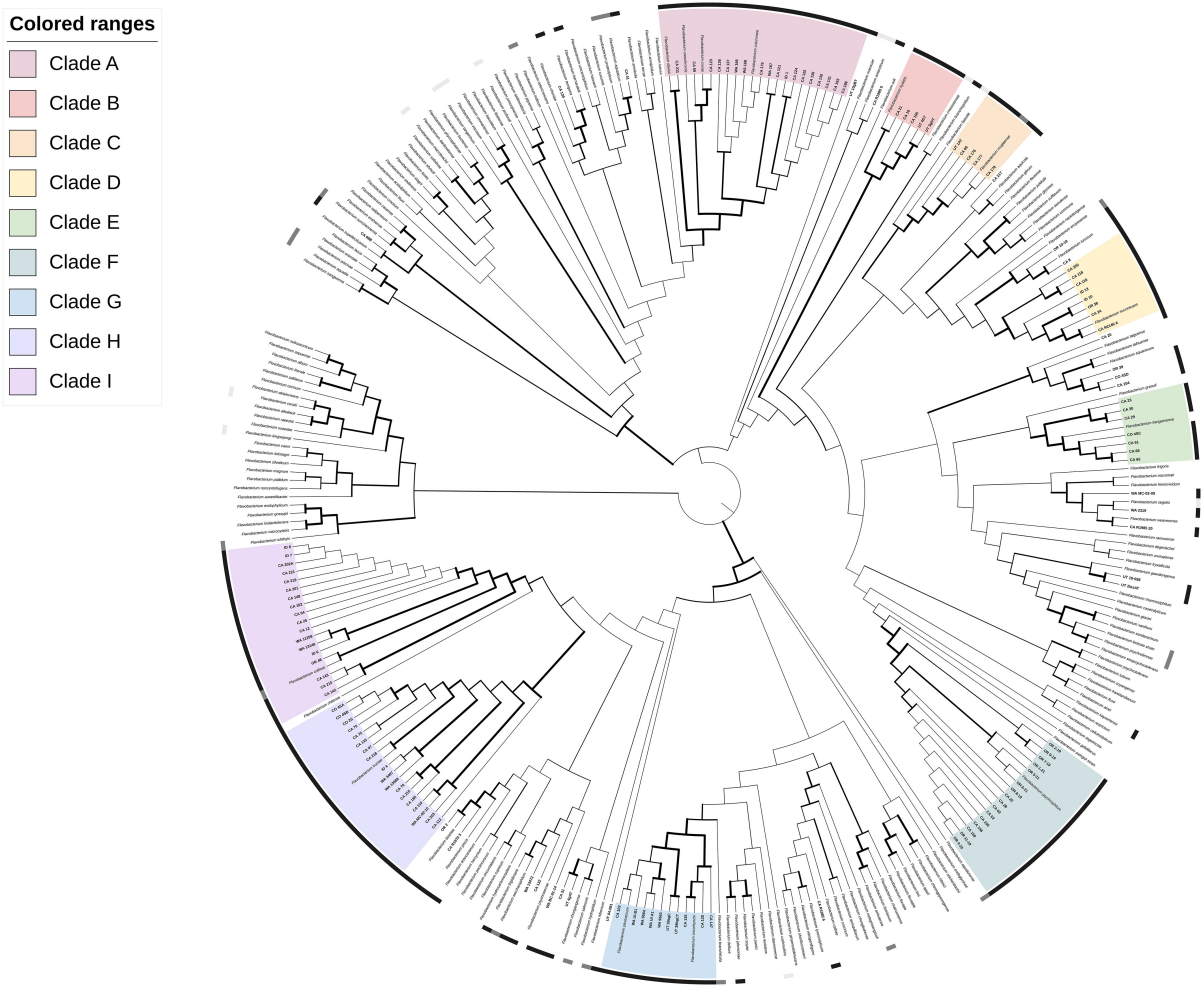
Phylogenetic relationships of 131 *Flavobacterium* spp. isolates recovered from diseased fishes in the Western US to 178 formally described *Flavobacterium* spp., based on partial *gyrB* gene sequences. The dendrogram was generated using the Maximum-Likelihood method and General Time Reversible model with a Gamma distribution allowing for evolutionarily invariable sites (GTR+G+I) and 1000 pseudoreplicates. Branch width is proportional to bootstrap support with increasing thickness from 0-1. Color strips indicate host relationship: black – fish disease associated, dark grey – fish associated, light gray – non-fish animal associated. Range color indicates clade: A, pink; B, red; C, orange; D, yellow; E, light green; F, teal; G, light blue; H, dark blue; I, purple. An interactive version of this tree with annotations, branch lengths and other features can be accessed at https://itol.embl.de/tree/3420946248377721671151594.

Phylogenetic analysis by the gyrase B gene placed the 131 *Flavobacterium* isolates into 9 major clades (A-I) consisting of at least five clinical isolates with high (≥ 70%) bootstrap support (BsS; Hillis and Bull, 1993) as well as several smaller groups of less than 5 isolates, and single lineages with, or without, published strains (**Figure 1**; https://itol.embl.de/tree/3420946248377721671151594). All clades contained isolates from more than one state. Clade A and F represented established fish pathogens, the columnaris causing bacteria (CCB; LaFrentz et al., 2022) and *F. psychrophilum* groups, respectively. Clade A (96.2% BsS) included 15 isolates from diverse fish hosts in CA, ID, and WA and *F. columnare, F. covae, F. davisii* and *F. oreochromis*. Isolate CA 211 clustered with *F. davisii*, and CA 59 and CA 123 with *F. covae.* All other isolates grouped closest to *F. columnare.* These groupings agreed with the 16S analysis, except for CA 59, an isolate from a chocolate cichlid (*H. temporalis*) that was most related to the type-strain of *F. inkyongense* (99.9% PI; **Supplementary Table 3**). Clade F (100% BsS) contained 16 salmonid isolates from OR and CA with the type-strain of *F. psychrophilum* from Coho salmon (*Oncorhynchus kisutch*). Isolates shared 99.2-100% PI with this type-strain in the 16S rRNA gene fragment.

Of the atypical fish disease-associated clades, Clade B (97.8% BsS) contained *F. hydatis*, previously isolated from diseased fish, and five UT and CA salmonid isolates. There were at least two subgroupings within the clade: the *F. hydatis* type-strain with CA 21 (73.9% BsS), and CA 16 with CA 155, UT 4DT and UT 3gHT (97.8% BsS). Clade C (100% BsS) was a well-defined group of salmonid isolates with high similarity to the *F. muglaense* type-strain from rainbow trout (*O. mykiss*). Clade D (100% BsS) contained isolates from salmonids, sturgeon and tilapia in CA, ID, and OR, along with the type-strain of *F. succinicans* from Chinook salmon (*Oncorhynchus tshawytscha*). The phylogenetic topography and 16S rRNA analysis of isolates in this clade suggest it consists of two to three species, including *F. succinicans.* Clade E (100% BsS) contains the type-strain of *F. tiangeerense* and clinical isolates from CA and CO. Two subgroups are evident: CA 23, CA 29, and CA 30 (95.4% BsS) and *F. tiangeerense*, CO 4SC, CA 61, CA 65 and CA 68 (99.9% BsS). Clade G (100% BsS) similarly contains two well-supported groups, with an established species in each. Group G1 (100% BsS) contains *F. oncorhynchi-*like isolates from koi and salmon in CA. Group G2 (100% BsS) contains *F. plurextorum-*like strains from salmonids in CA, WA and UT. The remaining atypical *Flavobacterium* clades were large groups of 18 isolates each. Clade H (100% BsS) consisted of isolates from salmonids and *Gambusia* in CA, CO, ID and WA, with the type-strain of *F. tructae* from a fish farm in Spain (Kämpfer et al., 2020). The type-strain formed an internal subgrouping with 8 of the 18 isolates (84.4% BsS) but there were additional potential subdivisions between isolates. Clade I (99.9% BsS) contained salmonid and sturgeon isolates from CA, OR and WA with the type-strain of *F. collinsii* from rainbow trout (*O. mykiss*) in Spain (Zamora et al., 2013b). Isolates CA 142, CA 143 and CA 210 formed a separate group (I1; 100% BsS) from the rest of the isolates and the type-strain (I2; 99.9% BsS).

Twenty-six isolates did not fall into a defined “major clade.” Isolates CA 104, CO-4SD, CA 39 and CA 20 formed a small group (100% BsS) by *gyrB* analysis that was not closely related to any reference strain (**Figure 1**). Isolate CA 41 clustered with *F. aquaticum* (BsS 100%) and isolates OR 2 and CA R19703 clustered with *F. bizetiae* from diseased freshwater fish in Canada (BsS 100%). Isolates CA 8 and OR 10-19 grouped with *F. erciyesense* and *F. turcicum* (100% BsS), which have been reported from diseased fish (**Table 1**). CA 139 was most closely related to *F. bernardetii* from rainbow trout (*O. mykiss*) and *F. terrigena* from soil (100% BsS). The remaining isolates were not well-resolved in the phylogeny.

#### 3.2.2 Chryseobacterium

**Figure 2:**
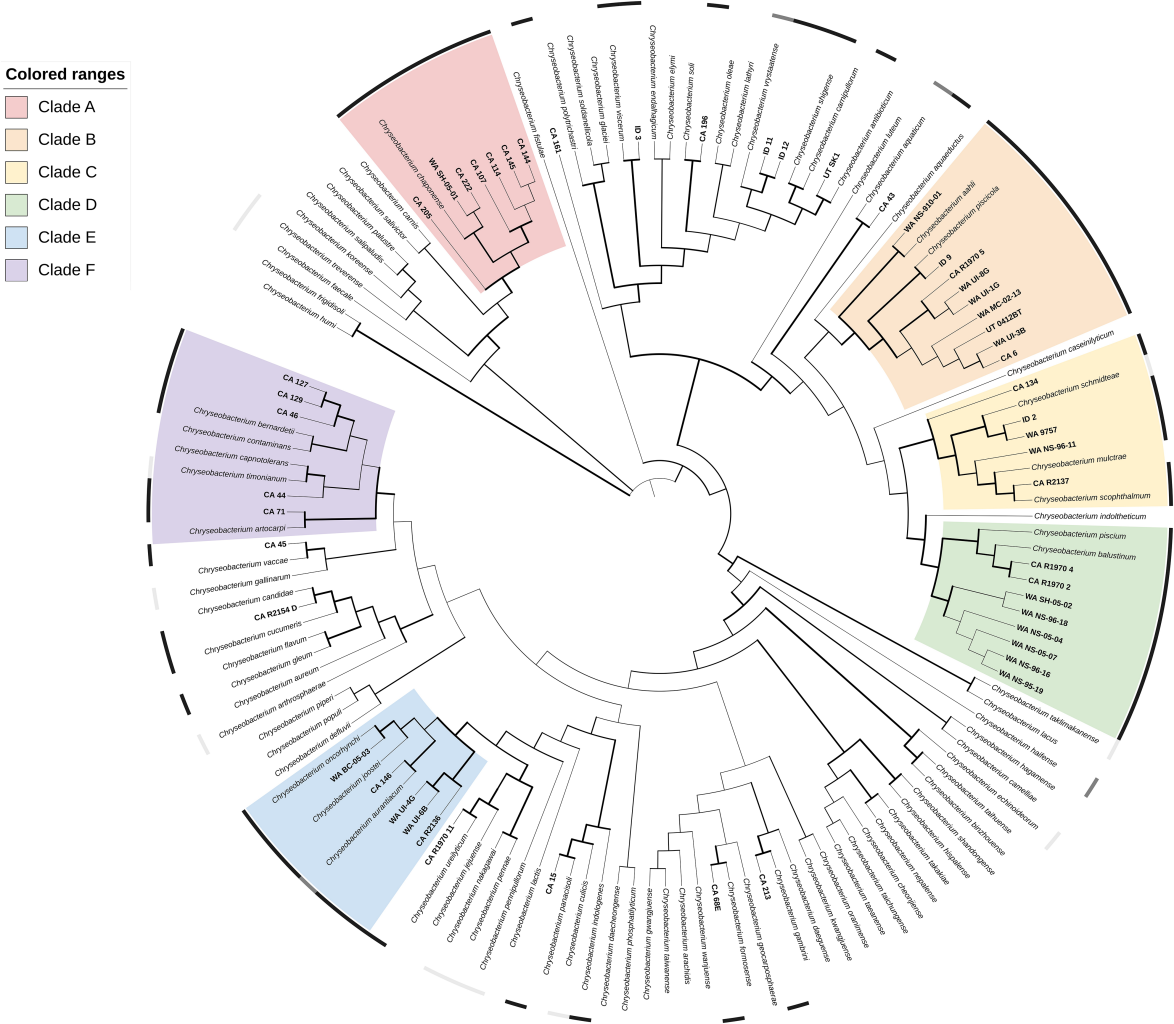
Phylogenetic relationships of 53 *Chryseobacterium* spp. isolates recovered from diseased fishes in the Western US to 94 formally described *Chryseobacterium* spp, based on partial *gyrB* gene sequences. The dendrogram was generated using the Maximum-Likelihood method and General Time Reversible model with a Gamma distribution allowing for evolutionarily invariable sites (GTR+G+I) and 1000 pseudoreplicates. Branch width is proportional to bootstrap support with increasing thickness from 0-1. Color strips indicate host relationship: black – fish disease associated, dark grey – fish associated, light gray – non-fish animal associated. Range color indicates clade: A, red; B, orange; C, yellow; D, green; E, blue; F, purple. An interactive version of this tree can be accessed at https://itol.embl.de/tree/31386026428981670886889.

Phylogenetic analysis through the gyrase B gene placed the 52 *Chryseobacterium* isolates into six major clades of more than five isolates with high (≥ 70%) bootstrap support (A-F), as well as several smaller lineages with, or without, related reference strains (**Figure 2**; https://itol.embl.de/tree/31386026428981670886889). Clade A (100% BsS) consisted of a single type-strain, *C. chaponense*, and seven isolates from *Oncorhynchus mykiss* varieties in CA and WA. Clade B (96.3% BsS) contained two species associated with fish disease, *C. aahli* and *C. piscicola*, and nine salmonid isolates from CA, WA, and ID. The *C. aahli* reference strain and WA NS-910-01 grouped together (91.1% BsS) and the remainder with *C. piscicola* (100% BsS). Clade C contained three type-strains, but only one from a species associated with fish disease – *C. scopthalmum.* Isolate ID 2 and WA 9757 grouped with *C. schmidteae* from a planarian (99.9% BsS) and WA NS-96-11 and CA R2137 loosely with *C. scopthalmum* and *C. multrae* (56.5% BsS). Isolate CA 134 branched off from the other strains at an earlier node. Clade D contained eight salmonid isolates from CA and WA and the type-strains from *C. piscium* and *C. balustinum.* The CA isolates were more closely related to the type-strains (D1; 97.5% BsS) than the WA strains, which formed their own subgroup (D2; 100% BsS). Clade E had 3 subgroups. Isolates CA R2136, WA UI-6B and WA UI-4G grouped without a type-strain (100% BsS), while CA 146 grouped with *C. aurantiacum* (88.5% BsS) and WA BC-05-03 with C. *oncorhynchi* (100% BsS). Clade F (97.1% BsS) contained five isolates and an equal number of type-strains from fish, human and environmental associated species. Within the clade, CA 46, 129 and 127 formed a subgroup with 99.9% bootstrap support. Twelve *Chryseobacterium* isolates did not fall into a major clade, though most branched closely (100% BsS) to a type-strain: CA 15 with *C. panacisoli*, CA 43 with *C. aquaticum*, CA 45 with *C. vaccae*, CA 196 with *C. soli*, CA 213 with *C. gambrini*, CA R1970 11 with *C. ureilyticum*, CA R2154 D with *C. candidae*, UT SK1 with *C. antibioticum*, and ID 3 with *C. viscerum*, The remainder of isolates were not well-resolved.

### 3.3 Phenotypic characterization

All tested isolates were gram-negative, catalase and oxidase positive rods. The representative *Chryseobacterium* isolates were all small, short and rounded. *Flavobacterium* isolates displayed higher morphological diversity, ranging from the long thin rods characteristic of *F. columnare* (LaFrentz et al., 2022) to shorter rounded rods, as observed with the Clade B isolate UT 3gHT (**Supplementary Figures 1, 2**). While all submitted isolates demonstrated yellow pigmentation, there were observed differences in hue. *Flavobacterium* exhibited either bright yellow (Clade A), gold (B, C, I), or translucent gold colonies (D-H) colonies on TYES. *Chryseobacterium* isolates displayed pale yellow (A), green-yellow (B) or gold (C-F) colonies (**Table 4** and **Supplementary Figures 1, 2**), although some isolates outside the major clades had orange and yellow-gray pigmentation. After 72 h of incubation at 20°C, *Chryseobacterium* formed smooth and rounded colonies of 0.25-1.25mm in diameter while *Flavobacterium* isolates either showed similar small, round morphologies or displayed spreading rhizoid colonies reaching ~4-7mm. All representative *Chryseobacterium* isolates tested grew on blood agar (BA), with α- or γ- hemolysis. The *F. columnare* and *F. psychrophilum* isolates tested, ID 1 and OR 1-21 respectively, did not grow on BA, nor did Clade E isolate CO 4SC. The remainder of tested isolates from the major clades were capable of growing on BA and were either α- or γ-hemolytic.

**Table 4:** Morphological characteristics of representative *Flavobacterium* and *Chryseobacterium* isolates from each major *gyrB* phylogenetic clade (**Figures 1**, **2**).

Isolate	*gyrB* Clade	Closest 16S hit	Colony phenotype	Size (mm)	Hemolysis^1^	Gram-stain	Catalase	Oxidase
ID 1	**A**	*F. columnare*	Rough, bright yellow	0.25-0.5	N/A	–	+	+
UT 3gHT	**B**	*F. branchiarum*	Spreading rhizoid, gold	4.0-7.0	α(+)	–	+	+
UT 1AT	**C**	*F. muglaense*	Gold-yellow, round	0.5-1.0	α(+)	–	+	+
ID 10	**D**	*F. succinicans*	Translucent, gold-yellow, round	0.5-1.25	γ(-)	–	+	+
CO 4SC	**E**	*F. taihuense*	Translucent gold-yellow, round	0.75-1.25	N/A	–	+	+
OR 1-21	**F**	*F. psycrophilum*	Translucent gold-yellow, round	0.25-0.5	N/A	–	+	+
UT 39agC	**G**	*F. plurextorum*	Spreading rhizoid, gold center with translucent edges	4.0-6.5	α(+)	–	+	+
CO 2S	**H**	*F. tructae*	Spreading rhizoid, gold center with translucent edges	5.0-7.0	α(+)	–	+	+
ID 7	**I**	*F. collinsii*	Spreading rhizoid, gold	5.0-6.0	α(+/-)	–	+	+
CA 144	**A**	*C. chaponense*	Pale yellow, round	0.25-1.0	γ(-)	–	+	+
WA UI-1G	**B**	*C. piscicola*	Green-yellow, round	0.5-1.0	α(+)	–	+	+
ID 2	**C**	*C. scopthalmum*	Gold-yellow, moist and round	1.5-2.0	α(+/-)	–	+	+
WA NS-05-04	**D**	*C. salvictor*	Gold-yellow, round	0.25-0.75	γ(-)	–	+	+
CA R2136	**E**	*C. oncorhynchi*	Gold-yellow, round	1.0-1.25	α(++)	–	+	+
CA 127	**F**	*C. indologenes*	Gold-yellow, round	1.0-1.5	α(++)	–	+	+

^1^Extent of hemolysis is indicated by type (α or γ) and qualitatively defined zone of hemolysis (+/-): tight ring,(+) moderate ring, (++) broad ring. N/A indicates no growth on blood agar.

### 3.4 Antimicrobial susceptibility profiling

There were trends in MIC profiles related to antimicrobial type and clade (**Table 5**). All clades in both the atypical *Flavobacterium* and *Chryseobacterium* genera had at least one tested isolate (total 33/39) with an MIC above the established *F. psychrophilum* breakpoint for oxytetracycline (CLSI, 2020). The MICs for florfenicol were also above the *F. psychrophilum* breakpoint for just over half of the tested isolates in each genus (24/39). A majority of isolates exhibited growth in the highest tested concentrations of cetifour (32/39), erythromycin (27/39), sulphadimethoxine (26/39) and penicillin (24/39) and MICs at the upper ranges of amoxicillin and spectinomycin. The MICs were generally, but not unanimously, lower for streptomycin. *Flavobacterium* isolates exhibited higher tolerance for trimethoprim/sulfamethoxazole, while *Chryseobacterium* isolates grew in higher concentrations of clindamycin. Within genera, *Flavobacterium* Clades B and G-I displayed overall comparably high MICs for florfenicol, trimethoprim/sulfamethoxazole, enrofloxacin, gentamicin, neomycin, erythromycin, amoxicillin, spectinomycin, sulphadimethoxine, sulphathiazole, and tylosin tartrate. *Chryseobacterium* clades E and F showed higher relative MICs for florfenicol, enrofloxacin, gentamicin, neomycin, amoxicillin, spectinomycin, sulphathiazole, streptomycin, novobiocin, tylosin tartrate and clindamycin. *Chryseobacterium* had overall a greater number of isolates with MICs exceeding testing concentration ranges.

**Table 5:** Antimicrobial susceptibility profiles of representative atypical *Flavobacterium* (CA 16-ID 7) and *Chryseobacterium* (CA 144-CA 44) strains.

Strain	Clade	Oxytetracycline	Florfenicol	Enrofloxacin	Trimethoprim/ Sulfamethoxazole	Gentamicin	Ceftiofur	Neomycin	Erythromycin	Tetracycline	Amoxicillin	Spectinomycin	Sulphadimethoxine	Sulphathiazole	Penicillin	Streptomycin	Novobiocin	Tylosin tartrate	Clindamycin
CA 16	**B**	2	> 8	0.25	1/19	> 8	> 4	8	> 4	2	> 16	> 64	> 256	> 256	> 8	64	> 4	> 20	< 0.5
CA 21	**B**	2	> 8	0.25	> 2/38	> 8	> 4	32	> 4	2	> 16	> 64	> 256	> 256	> 8	32	> 4	> 20	< 0.5
UT 3gHT	**B**	4	> 8	0.25	> 2/38	> 8	> 4	32	> 4	4	> 16	> 64	> 256	> 256	> 8	64	4	> 20	< 0.5
CA 66	**C**	> 8	< 1	< 0.12	< 0.5/9.5	1	> 4	< 2	0.25	> 8	0.5	16	< 32	< 32	8	< 8	2	< 2.5	< 0.5
UT 1AT	**C**	> 8	< 1	< 0.12	1/19	2	> 4	< 2	0.25	> 8	0.5	32	64	< 32	> 8	< 8	4	< 2.5	< 0.5
CA 178	**C**	> 8	< 1	< 0.12	< 0.5/9.5	1	> 4	< 2	> 4	> 8	< 0.25	16	< 32	< 32	8	16	2	< 2.5	< 0.5
CA 24	**D**	4	< 1	< 0.12	< 0.5/9.5	1	> 4	< 2	2	2	1	64	< 32	< 32	1	< 8	2	< 2.5	< 0.5
ID 10	**D**	2	< 1	< 0.12	< 0.5/9.5	1	> 4	< 2	4	2	0.5	16	< 32	< 32	4	< 8	2	10	< 0.5
CA R2146 A	**D**	< 0.25	< 1	< 0.12	< 0.5/9.5	1	> 4	< 2	1	< 0.25	< 0.25	32	< 32	< 32	0.5	< 8	2	5	< 0.5
CA 30	**E**	< 0.25	< 1	< 0.12	< 0.5/9.5	< 0.5	2	< 2	0.25	< 0.25	< 0.25	32	< 32	< 32	0.25	0.25	1	< 2.5	< 0.5
CA 61	**E**	8	< 1	< 0.12	< 0.5/9.5	< 0.5	4	< 2	0.5	4	< 0.25	16	< 32	< 32	0.5	< 8	1	< 2.5	< 0.5
CO 4SC	**E**	< 0.25	< 1	< 0.12	< 0.5/9.5	< 0.5	> 4	< 2	2	< 0.25	< 0.25	16	< 32	< 32	2	< 8	2	< 2.5	< 0.5
UT 39agC	**G**	2	4	0.25	> 2/38	> 8	> 4	16	> 4	4	16	> 64	> 256	> 256	> 8	32	2	> 20	1
CA 135	**G**	2	4	0.5	> 2/38	> 8	> 4	16	> 4	4	16	> 64	> 256	> 256	> 8	32	< 0.5	> 20	< 0.5
WA UI-A1	**G**	4	4	0.25	> 2/38	> 8	> 4	8	> 4	4	1	> 64	> 256	256	4	16	2	> 20	< 0.5
CA 203	**H**	2	8	0.25	> 2/38	> 8	> 4	16	> 4	4	16	> 64	> 256	256	> 8	256	0.5	< 2.5	< 0.5
CO 2S	**H**	0.5	2	< 0.12	2/38.	8	> 4	> 32	4	1	16	64	> 256	< 32	> 8	< 8	< 0.5	20	< 0.5
WA MC-02-10	**H**	2	4	< 0.12	> 2/38	> 8	> 4	16	> 4	4	16	64	> 256	256	> 8	128	< 0.5	> 20	< 0.5
WA 11299	**I**	4	> 8	0.25	> 2/38	> 8	> 4	16	> 4	8	16	> 64	> 256	256	> 8	64	< 0.5	> 20	1
CA 143	**I**	1	4	< 0.12	> 2/38	> 8	> 4	32	> 4	1	16	> 64	> 256	256	> 8	< 8	< 0.5	> 20	< 0.5
ID 7	**I**	2	4	0.25	1/19	> 8	> 4	8	> 4	4	16	> 64	< 32	128	> 8	32	< 0.5	< 2.5	< 0.5
CA 144	**A**	8	< 1	< 0.12	0.5/9.5	< 0.5	> 4	< 2	4	4	16	16	64	128	> 8	< 8	< 0.5	< 2.5	< 0.5
CA 205	**A**	> 8	< 1	< 0.12	< 0.5/9.5	1	4	< 2	> 4	> 8	8	32	> 256	256	8	< 8	< 0.5	< 2.5	< 0.5
WA SH-05-01	**A**	> 8	< 1	< 0.12	< 0.5/9.5	< 0.5	2	< 2	1	8	16	< 8	> 256	< 32	> 8	< 8	< 0.5	< 2.5	< 0.5
WA UI-1G	**B**	0.5	2	< 0.12	< 0.5/9.5	1	> 4	< 2	> 4	0.5	8	32	< 32	> 256	8	< 8	< 0.5	5	< 0.5
WA UI-3B	**B**	< 0.25	2	< 0.12	< 0.5/9.5	1	4	< 2	> 4	< 0.25	8	32	< 32	256	8	< 8	< 0.5	< 2.5	< 0.5
WA MC-02-13	**B**	< 0.25	2	< 0.12	< 0.5/9.5	2	> 4	< 2	> 4	1	8	64	> 256	256	8	< 8	< 0.5	5	< 0.5
WA 9757	**C**	2	8	< 0.12	< 0.5/9.5	4	4	4	> 4	4	> 16	64	> 256	256	> 8	64	1	10	4
CA 134	**C**	2	4	> 2	< 0.5/9.5	4	> 4	4	> 4	4	> 16	> 64	> 256	> 256	> 8	32	< 0.5	20	> 4
ID 2	**C**	1	2	< 0.12	< 0.5/9.5	2	> 4	4	> 4	2	> 16	64	> 256	256	> 8	< 8	< 0.5	10	1
WA NS-05-04	**D**	< 0.25	2	< 0.12	< 0.5/9.5	2	> 4	< 2	4	< 0.25	2	64	> 256	64	4	< 8	4	< 2.5	< 0.5
WA NS-95-19	**D**	> 8	< 1	< 0.12	< 0.5/9.5	1	2	< 2	> 4	> 8	4	16	> 256	256	4	< 8	< 0.5	< 2.5	< 0.5
WA SH-05-02	**D**	> 8	< 1	< 0.12	< 0.5/9.5	< 0.5	> 4	< 2	1	8	16	< 8	> 256	256	> 8	< 8	< 0.5	< 2.5	< 0.5
CA R2136	**E**	> 8	8	2	1/19	> 8	> 4	> 32	> 4	> 8	> 16	> 64	> 256	> 256	> 8	> 1024	2	> 20	> 4
WA UI-4G	**E**	> 8	8	1	< 0.5/9.5	> 8	> 4	> 32	> 4	> 8	> 16	> 64	> 256	> 256	> 8	128	0.5	> 20	> 4
WA UI-6B	**E**	> 8	8	0.5	< 0.5/9.5	> 8	> 4	> 32	> 4	> 8	> 16	> 64	> 256	> 256	> 8	128	< 0.5	> 20	> 4
CA 127	**F**	> 8	> 8	1	1/19	> 8	> 4	> 32	> 4	> 8	> 16	> 64	> 256	> 256	> 8	128	2	> 20	> 4
CA 129	**F**	> 8	> 8	0.5	< 0.5/9.5	> 8	> 4	> 32	> 4	> 8	> 16	> 64	> 256	> 256	> 8	256	2	> 20	> 4
CA 44	**F**	8	4	0.5	1/19	> 8	> 4	32	> 4	8	> 16	> 64	> 256	> 256	> 8	512	< 0.5	> 20	> 4
Flavobacterium psycrophilum	WT	≤ 0.12	≤ 2	≤ 0.03	N/A	N/A	N/A	N/A	≤ 8	N/A	N/A	N/A	N/A	N/A	N/A	N/A	N/A	N/A	N/A
	NWT	≥ 0.25	≥ 4	≥ 0.06	N/A	N/A	N/A	N/A	≥ 16	N/A	N/A	N/A	N/A	N/A	N/A	N/A	N/A	N/A	N/A

Clade denotes phylogenetic grouping by *gyrB* analysis (**Figures 1** and **2**). Values in bold represent minimal inhibitory concentrations (MICs) higher than established wild-type (WT) MICs for *F. Psychrophilum* for the relevant antimicrobials. Color shading denotes scaling from MICs exceeding the tested range (dark blue) to those below the tested range (white)."N/A" means "not applicable".

## 4 Discussion

Flavobacteria are widely recognized as important members of environmental and piscine microbial communities, but the nuances to their taxonomic placements and their contribution to disease remain enigmatic. With ongoing refinement in the methods available for bacterial characterization, substantial readjustments to the structure of Flavobacteriales have occurred at every scale. Following the formal description and delineation of the genus *Chryseobacterium* from *Flavobacterium* in the early 2000s, the taxonomy of the genus has been further revised and moved from *Flavobacteriaceae* to a new family, *Weeksellaceae* (Bernardet et al., 2006; García-López et al., 2019). Within both genera, new species are frequently described, and existing species either subsumed into synonymous species or split. Even the most well-studied flavobacteria are subject to changes under new scrutiny, with the recent division of *Flavobacterium columnare* genetic groups into four distinct species (LaFrentz et al., 2022). This constant upheaval requires constant vigilance to remain aligned with current taxonomic structures, complicating identification of new isolates by comparison to historical strains. In addition to discrepancies in strain names in databases and the literature, conclusive classification of flavobacteria remains difficult by standard diagnostic methods (Loch and Faisal, 2015a).

The most common method for bacterial molecular identification is sequencing of the 16S rRNA gene, with the locus having been successfully used in conjunction with other markers for the delineation of many novel flavobacterial taxa (Bernardet et al., 2002). However, use of this locus in flavobacterial typing is also accompanied by some pitfalls. The genomic 16S rRNA gene copy number ranges from 1-13 in flavobacterial species (Stoddard et al., 2015), and there is often high interspecies sequence similarity (Loch and Faisal, 2015a). Conventionally, the cut-off indicating speciation in the 16S rRNA gene is 97% pairwise identity between an isolate and the most closely related reference species (Edgar, 2018). However, a higher threshold of 98.6-99% has been proposed to improve discrimination of bacterial species with high genetic homogeneity in the gene, such as with *Flavobacterium* and *Chryseobacterium* (Stackebrandt and Ebers, 2006; Edgar, 2018). Even using this elevated threshold, there are abundant examples of isolates with high 16S rRNA gene similarity to a type species that may group disparately phylogenetically, or be assigned as a novel or separate species by subsequent polyphasic analysis (Zamora et al., 2012a; Loch et al., 2013; Loch and Faisal, 2015b; Loch and Faisal, 2016a; Verner-Jeffreys et al., 2017; Loch and Faisal, 2018). The gene for DNA gyrase subunit B, *gyrB*, has been suggested as an alternative or supplementary housekeeping gene for bacterial genotyping (Wang et al., 2007; Galloway-Peña et al., 2014; Reichley et al., 2017). As a single copy gene with a faster evolutionary rate than the 16S rRNA gene, *gyrB* often offers improved and more accurate discriminatory power. Reflecting this, there was high homogeneity in the 16S rRNA gene fragment between type-strains and isolates in both genera, with an average pairwise identity of 92.5% across 400 strains in *Flavobacterium* and 94.8% across 189 *Chryseobacterium* strains. While a comparable analysis cannot be run for the *gyrB* gene, as many type-strains do not have published *gyrB* sequences available, running alignments using the subset of type-strains with whole genomes available shows the average PI for the 16S rRNA gene remains similar, with an average of 93.4% pairwise identity across 309 *Flavobacterium* strains and 94.9% across 144 *Chryseobacterium* strains. Comparison of the *gyrB* fragment among those same strains reveals higher inter-strain heterogeneity, with 79.1% PI across the *Flavobacterium* strains and 80.6% across *Chryseobacterium*. The maximum likelihood trees generated by *gyrB* analysis subsequently offered higher resolution and more defined clades with higher bootstrap support than those generated by equivalent methods using the 16S rRNA gene (**Figures 1**, **2** and **Supplementary Figures 3, 4**).

We found a diverse assemblage of flavobacteria associated with aquaculture and fish disease in the Western United States. The 131 *Flavobacterium* spp. and 53 *Chryseobacterium* spp. were recovered from wild fish, ornamental fish, hatchery raised, or commercially produced species. Salmonids, in particular rainbow trout (*O. mykiss*) varieties, were most represented, consistent with regional aquaculture activities and historical epidemiological trends for flavobacterial disease (Wahli and Madsen, 2018; United States Department of Agriculture, 2019). Most isolates (125/183) shared high (≥ 99%) similarity to one or more reference species previously linked to fish or fish disease (**Supplementary Table 1**) either in the Great Lakes region of the United States, or in South America, Europe, Asia, or Africa (Loch and Faisal, 2015a). Though conclusive confirmation of isolate identities by whole genome analysis is warranted, the results of this study expand the geographic and host ranges of established species, the majority of which have never been reported in the Western United States.

Phylogenetic analysis using the *gyrB* gene determined the presence of multiple genetic clusters in both genera associated with fish disease (**Figures 1**, **2**). The majority of isolates (151/184) collected from clinically affected fish in the Western United States fell into one of 15 major clades within these larger clusters. Genetic groups were not clearly based on geographic origin, and isolates from the same state were phylogenetically diverse. *Flavobacterium* isolates formed nine clades (A-I), including isolates closely related to the well-known flavobacterial pathogens – *F. psychrophilum* and the CCB. No isolates were identified as *F. branchiophilum.* Clade F (*F. psychrophilum-*like) isolates were largely homogenous and were all isolated from salmonid species, consistent with previous reports. The CCB group had diverse representation by different fish hosts, with 10 different fish types from 6 different genera. The CCB species have shown some patterns in host specificity in previous studies (LaFrentz et al., 2021). *Flavobacterium oreochromis*, associated with tilapia, has not been reported in the US and was not identified in this study. The cichlid isolate in the CCB group (CA 59) clustered with *F. covae* by *gyrB* analysis, a species previously tied to catfish disease, but shared 99.9% PI with related species *F. inkyongense* in the 16S rRNA gene fragment. *Flavobacterium inkyongense* did not have a reference genome available and was therefore not included in phylogenetic analysis. Isolate CA 123 from zebrafish (*D. reino*) also grouped with *F. covae* and shared high (99.9%) 16S rRNA gene similarity with the type-strain. *Flavobacterium davisii* has been recovered from columnaris cases in rainbow and steelhead trout in the US (Sebastião et al., 2019a), and similarly was most closely related to rainbow trout (*O. mykiss*) isolate CA 211 by both 16S and *gyrB* analyses. *Flavobacterium columnare* is also associated with salmonids, and the phylogenetic subgroup in this study included mostly salmonid strains, alongside two koi (*C. rubrofuscus*) isolates.

The atypical clusters B-E and G-I contained species of growing interest to aquaculture and fish health, along with potentially novel agents of fish disease. For example, Clade B, contained the type-strain of *F. hydatis*, which has been historically associated with fish disease (Strohl and Tait, 1978; Bernardet and Bowman, 2006; Mantareva et al., 2022). However, the five clinical isolates in the group did not share high 16S rRNA gene sequence similarity to *F. hydatis* nor any other type-strain (**Supplementary Table 3**). It is possible they represent up to three new species. In previous studies in other regions of the US, isolates most closely related to *F. hydatis*, but similarly falling below the 99% 16S rRNA gene pairwise identity threshold, have been recovered (Loch et al., 2013; Loch and Faisal, 2014a). It would be interesting to compare these isolates more closely. Clade E was also made up of isolates all below the 99% 16S rRNA gene threshold, with the type-strain *F. tiangeerense* (Xin et al., 2009). Similarly, to *F. hydatis*, other studies in the US have recovered fish isolates moderately similar to this species (Loch et al., 2013; Loch and Faisal, 2014a; Loch and Faisal, 2018), suggesting additional yet-undefined agents associated with flavobacterial disease.

Phylogenetic subgroupings observed in the other clades suggest that additional novel species or subspecies are likely. Clade D contains *F. succinicans*, previously implicated in fish disease (Anderson and Ordal, 1961; Good et al., 2015; Adamek et al., 2018; Loch and Faisal, 2018; Einarsdottir et al., 2020), but isolates ranged in pairwise 16S rRNA gene similarity from 98.2-99.9%. The type-strain in Clade H, *F. tructae*, is one of the few atypical *Flavobacterium* with demonstrated pathogenicity in fish (Zamora et al., 2013a; Loch and Faisal, 2016b). *Flavobacterium tructae* has only been reported in salmonids, but Clade H also included an isolate from *Gambusia* with high 16S rRNA gene similarity (99.6% PI). Six out of 18 isolates in Clade H, however, showed low pairwise identity to *F. tructae*, and higher similarities to *F. salmonis* (98.4-99.1%) or *F. chungangense* (98%). Three isolates from Clade I also shared more sequence identity (98.8% PI) with *F. salmonis* in the 16S rRNA gene. The remainder of Clade I isolates were most related (99.7-100% PI) to *F. collinsii*, which has not been clearly linked to fish disease nor identified in the US. The type-strain of *F. collinsii* was recovered from healthy rainbow trout (*O. mykiss*) (Zamora et al., 2013b), and a recently released pre-print recovered the species from a fish that died without clinical signs or apparent lesions (Lee et al., 2022). Attempts to fulfill Koch’s postulates were unsuccessful, although the bacteria was recovered from internal tissues of sampled, apparently healthy survivors. Nevertheless, the frequency of *F. collinsii* isolations from diseased fishes in this study warrants further comparisons of strains under different challenge models.

The most well-defined clades outside of Clade A (CCB) and Clade F (*F. psychrophilum)* were Clades C and G. Clade C contained a type-strain from a species associated only with healthy rainbow trout outside of the US – *F. muglaense* (Duman et al., 2021). Salmonid isolates from CA and UT shared 99-100% 16S rRNA gene similarity to the type-strain, and the phylogenetic tree topography indicates high similarity within the *gyrB* gene as well. Recently, an isolate sharing 100% sequence identity to *F. muglaense* across an ~ 1,100 bp stretch of the 16s rRNA gene was also recovered from the kidneys of diseased hatchery-raised rainbow trout (*O. mykiss*) in Michigan (Loch, unpublished), indicating it is a species of interest for aquaculture. Clade G included two subgroups defined by closely related species – *F. oncorhynchi* and *F. plurextorum*. Both species have been reported in association with salmonids and other species, in diseased or healthy farmed and wild fish (Zamora et al., 2012a; Loch et al., 2013; Zamora et al., 2013a; Loch and Faisal, 2014a; Loch and Faisal, 2018). *Flavobacterium oncorhynchi* has also been implicated in a fatal human fetal infection (Ferreira et al., 2022). Virulence in fish, however, has not been formally investigated for either species. Although these species share high genetic similarity, they have been confirmed as distinct, and 16S results also support delineation of the clade.

In addition to the major clades, there were single isolates and minor clades of interest. Isolate CA 139 was closely related to *F. bernardetii*, a recently described species from diseased rainbow trout (*O. mykiss*) that has not been reported in the United States (Saticioglu et al., 2021b). Isolate OR 10-19 and CA 8 grouped with *F. erciyesense* and *F. turcicum*, previously associated with fish disease in Turkey (Saticioglu, 2021; Saticioglu et al., 2021a), while CA 44 grouped with *F. aquaticum*, which has only been reported in healthy catla (*Catla catla*) in India (Verma and Rathore, 2015). Isolates CA R1970 3 and CA 2 branched with *F. bizetiae*, *F. araucananum* and *F. piscis*, which have all been linked to fish disease (**Table 1**). Two isolates also grouped weakly with the strains isolated from penguin feces or habitats, possibly indicating a dietary transmission from fish (**Figure 1**).

*Chryseobacterium* clades were somewhat less resolved compared to *Flavobacterium*, likely due to undersampling (**Figure 2**). In particular, Clade F was a group of five isolates and an equal number of type-strains. The *C. bernardetii* and *C. timonianum* type-strains are human clinical isolates (**Supplementary Table 2**), but *C. bernardetii* has also been recovered from diseased rainbow trout (*O. mykiss;* Saticioglu et al., 2021b) indicating a potential avenue for zoonotic transmission. *Chryseobacterium* spp. can be opportunistic human pathogens in invasive disease. *Chyrseobacterium indologenes* for example, has a wide host range that includes both fish and humans, though zoonotic transmission has not been reported (Hugo et al., 2019). Interestingly, while two Clade F isolates shared the highest 16S rRNA gene sequence similarity with *C. indologenes*, no isolates grouped closely with it in the *gyrB* tree. The 16S rRNA and whole genome (*gyrB*) gene sequences used in analysis were both from the *C. indologenes* type-strain, so this difference may be due to low resolution in 16S rRNA gene, or perhaps differences in published sequence assemblies.

All remaining *Chryseobacterium* clades (A-E) also had ties to fish disease. Clade A was a well-defined group of isolates collected from *O. mykiss* varieties and the type-strain of *C. chaponense*, also recovered from diseased rainbow trout (*O. mykiss*) (Kämpfer et al., 2011). A strain with high similarity to *C. chaponense* (> 99.6%) has been assessed for virulence in salmonids, and while it did not cause any mortalities, it did induce lesions, including melanosis, swollen congested liver, swollen enlarged spleen and mottled kidney (Loch and Faisal, 2015b). Clade B contained salmonid isolates and two species with confirmed piscine pathogenicity – *C. aahli* and *C. piscicola.* Isolate WA-NS-910-01 grouped closely with *C. aahli* by *gyrB* analysis but was not highly similar to any of the type-strains by 16S analysis (98.4% PI to *C. aquaticum*). The remaining isolates, however, shared high sequence similarity with the *C. piscicola* type-strain (99.6-100%). This species has been reported in outbreaks of disease in salmonids in Chile and recovered in the Great Lakes region (Ilardi et al., 2009; Ilardi et al., 2010; Loch et al., 2013; Loch and Faisal, 2015b). Clade C consisted of koi and salmonid isolates, with type-strains related to animals and animal products. *Chryseobacterium schmidteae* was collected from a planarian, and *C. mulctrae* from raw cow milk. *Chryseobacterium scophthalmum* is an established fish pathogen with demonstrated pathogenicity in more than one fish host (Mudarris and Austin, 1989; Mudarris et al., 1994; Shahi et al., 2018). Interestingly, while CA R2137 clustered closest to this species by *gyrB* analysis, ID 2 showed higher similarity by 16S rRNA gene alignment. The type-strains in Clade D - C*. balustinum* and *C. piscium –* represent species with experimental evidence suggesting pathogenic potential (**Table 2**). Chinook salmon (*O. tshawytscha*) isolates from California, CA R1970 2 and CA R1970 4 grouped closely to the type strains and were most related to *C. piscium* by 16S analysis (98.9% PI), but the WA isolates from rainbow trout (*O. mykiss*) formed a distinct subgroup. Finally, Clade E contained three piscine pathogenic species – *C. oncorhynchi, C. joostei*, and *C. aurantiacum –* but only the relationships to *C. oncorhynchi* and *C. joostei* were supported by the 16S results. Previously, *C. oncorhynchi* has been reported in rainbow trout (*O. mykiss*) and Siberian sturgeon (*Acipenser baerii*) (Zamora et al., 2012a; SuXu et al., 2019), but the clade included CA R2136 from tilapia (*Oreochromis* spp.) and CA 146 from koi (*C. rubrofuscus*). Outside of the major clades, several isolates still branched closely with fish associated species, such as *C. vrystaatense*, which has been recovered previously from healthy wild fish in the Great Lakes (Loch et al., 2013) and *C. aquaticum* from diseased fish cases (Bernardet et al., 2005; Loveland-Curtze et al., 2010; Hugo et al., 2019; Sebastião et al., 2019b; Saticioglu et al., 2021c)

The tangled taxonomy of Flavobacteriales and the lack of consistently effective challenge models have limited our understanding of the risk these atypical species pose to aquaculture. As such, the full characterization of species of interest and their virulence to fish hosts remain largely unexplored and are the subject of ongoing investigations. Preliminary results demonstrate notable variations in colony and cell morphology between study isolates, as well as in hemolytic profiles (**Table 4** and **Supplementary Figures 1, 2**), suggesting that the observed genetic diversity underlies important phenotypic and virulence differences. Additionally, there were 58 isolates below the 99% 16S rRNA gene sequence similarity threshold to any available type-strain, and a number of isolates that formed phylogenetic subgroupings distinct from their closest type species. These potentially represent new species or subspecies in the genera. Different strains within highly represented clades like *F. collinsii, F. tructae* or *C. chaponense* should also be assessed for any strain-specific trends in virulence using biologically relevant challenge models.

The high association of these flavobacterial groups with recent and historical outbreaks of disease remain suggestive of their pathogenic potential. It is likely that some of these atypical piscine flavobacteria represent opportunistic pathogens, but this does not diminish their potential importance to aquaculture. High intensity rearing systems favor conditions for opportunistic infections, and flavobacterial diseases are notoriously difficult to treat and control (Verner-Jeffreys et al., 2017; Kumru et al., 2020; Saticioglu et al., 2021c). Both *Chryseobacterium* and *Flavobacterium* display a propensity for resistance across antimicrobial classes. Though there is some evidence for horizontal transfer of resistance genes from other gram-negative bacteria, this elevated level of resistance is thought to be primarily intrinsic rather than anthropogenically driven (Verner-Jeffreys et al., 2017). Mechanisms may include non-specific efflux pumps, chromosomally linked extended-spectrum beta-lactamases, and alterations in DNA gyrase and topoisomerase genes (Michel et al., 2005; Chen et al., 2017; Verner-Jeffreys et al., 2017; Kumru et al., 2020; Saticioglu et al., 2021b; Saticioglu et al., 2021c). The representative isolates tested from each major clade reflected established trends, with generally high MICs across multiple antimicrobial types. Clades F and G in *Chryseobacterium* and B and G-I in *Flavobacterium* showed comparably higher MICs for at least 11/18 antimicrobials*. Flavobacterium hydatis* from clade B, *F. plurextorum* from clade G, and *F. chilense*, which branched closely with Clades H and I, were also reported in a previous study to have the highest number of antimicrobial resistance (AMR) genes out of 86 different *Flavobacterium* genomes investigated (Kumru et al., 2020). Clinical CLSI breakpoints are not established for any of the atypical flavobacteria, but at least one isolate in every clade demonstrated an MIC at or above the concentration required for resistance in *F. psychrophilum* for oxytetracycline and florfenicol, two of the three antimicrobials approved for use in aquaculture in the United States. Isolates from *Flavobacterium* Clade B/G-I also exceeded the testing range for trimethoprim/sulfamethoxazole, a potentiated sulfonamide, while almost all *Chryseobacterium* tested were inhibited by this antimicrobial at the lowest concentration. The elevated susceptibility of *Chryseobacterium* to trimethoprim/sulfamethoxazole is consistent with the literature but is not universal. A survey of 70 C*. aquaticum* isolates from diseased rainbow trout in Turkey found the average MIC for trimethoprim/sulfamethoxazole to be 2/38, the highest concentration tested in our study format (Saticioglu et al., 2021c).

High MICs indicate that treatment of emerging flavobacterial pathogens with approved antimicrobials may be unsuccessful. Preventing outbreaks is also difficult, as some *Flavobacterium* and *Chryseobacterium* spp. are ubiquitous in the environment and disinfection of fish eggs or tanks with common antimicrobials can be insufficient to eradicate the bacteria. Many flavobacteria form biofilms, and there is evidence that iodophor disinfection of salmon eggs may not eliminate some of the bacterial species identified in this study (Loch and Faisal, 2018). Successful vaccination against flavobacterial disease is another challenge complicated by the high diversity of piscine pathogenic species and the propensity for mixed species outbreaks. Recent studies with a live-attenuated vaccine against *F. psychrophilum* have shown some promising preliminary results for cross-protection and conserved antigens between *F. psychrophilum* and atypical *Flavobacterium* and *Chryseobacterium* isolates (Bruce et al., 2020), but there remains much work to be completed to reach targets for widespread protection.

Assembling the depth and breadth of information required to effectively control this complex group of diseases is a tremendous undertaking. The results of this study add another piece to our understanding of the flavobacteria contributing to fish disease, in an understudied, but crucial region for aquaculture. Isolates similar to fish pathogenic species identified in the Great Lakes region of the US and in South America, Europe, Africa and Asia were recovered in the Western United States, expanding the geographic, and in some cases, host range, of these atypical agents of flavobacterial disease. Continuing investigations stemming from this study, addressing isolate pathogenesis, ecology, and genetic and antigenic diversity, will create a more comprehensive picture of piscine flavobacterial disease in the United States, with improved recommendations for treatment and control.

## Data availability statement

Partial 16S rRNA and gyrase B gene sequences generated as part of this study were uploaded to GenBank, Accession numbers OQ546252-OQ546434 and OQ561006-OQ561188. All phylogenetic trees generated in this project can be accessed at https://itol.embl.de/shared/tiheckman under project “Atypical Flavobacteria in Aquaculture.

## Author contributions

KC, TH, TL, ES, FS, BV and ZY contributed to the conceptualization and design of the study. All authors contributed to the acquisition and characterization of bacterial isolates. TH, KM, EP, FS and ZY contributed to the sequencing of bacterial isolates. ZY and ES preformed MIC analyses. TH preformed genetic, phylogenetic, and phenotypic analyses and wrote the first draft of the manuscript. All authors contributed to the article and approved the submitted version.
