# Supplementary material for: Atypical flavobacteria recovered from diseased fish in the Western United States
Source: Front Cell Infect Microbiol. 2023 Apr 19;13:1149032. doi: 10.3389/fcimb.2023.1149032 (PMC10161732; doi:10.3389/fcimb.2023.1149032)
Supplement: Supplementary file 1 [file DataSheet_1.docx]

Supplementary Material

Atypical Flavobacteria Recovered from Diseased Fish in the Western United States

Taylor I. Heckman, Zeinab Yazdi, Eric K. Pomaranski, Fernanda de Alexandre Sebastião, Kaveramma Mukkatira, Brent M. Vuglar, Kenneth D. Cain, Thomas P. Loch, Esteban Soto*

*** Correspondence:** Corresponding Author: [sotomartinez@ucdavis.edu](mailto:sotomartinez@ucdavis.edu)

**STable 1:** *Flavobacterium* strains with publicly available whole genomes used in phylogenetic analysis. Reference genomes used when type-strain genomes were not available are indicated with an asterisk. Host association (fish or other animal) may not reflect the isolation source of the type-strain but subsequent published isolations.

|  | **Name** | **Accession** | **Source** | **Country** | **Collection date** |
| --- | --- | --- | --- | --- | --- |
| **Fish associated** | *Flavobacterium anhuiense* strain CGMCC 1.6859 | GCA_900101855.1 | Soil | China | Unk |
|  | *Flavobacterium aquaticum* strain CGMCC 1.12398 | [GCA_003259835.1](https://www.ncbi.nlm.nih.gov/data-hub/genome/GCA_003259835.1/) | Freshwater | India | Unk |
|  | *Flavobacterium aquatile* ATCC 11947 | GCA_002217235.1 | Deep well | UK | 1995 |
|  | *Flavobacterium aquidurense* strain DSM 18293 | GCA_002217195.1 | Spring water | Germany | Unk |
|  | *Flavobacterium araucananum* strain DSM 24704 | GCA_003148525.1 | Atlantic salmon (*Salmo salar*) | Chile | 2008 |
|  | *Flavobacterium bernardetii* strain F-372 | [GCA_011305415.1](https://www.ncbi.nlm.nih.gov/data-hub/genome/GCA_011305415.1/) | Rainbow trout (*Oncorhynchus mykiss*) | Turkey | 2017 |
|  | *Flavobacterium bizetiae* strain CIP 105534 | [GCA_904425415.1](https://www.ncbi.nlm.nih.gov/data-hub/genome/GCA_904425415.1/) | Diseased freshwater fish | Canada | Unk |
|  | *Flavobacterium branchiicola* strain CCM 9061* | [GCA_018383905.1](https://www.ncbi.nlm.nih.gov/data-hub/genome/GCA_018383905.1/) | Rainbow trout (*O. mykiss*) | Spain | 2009 |
|  | *Flavobacterium branchiophilum* ATCC 35036 | GCA_002530755.1 | Masu salmon (*Oncorhynchus masou*) | Japan | 1977 |
|  | *Flavobacterium chilense* strain DSM 24724 | GCA_900142685.1 | Rainbow trout (*O. mykiss*) | Chile | Unk |
|  | *Flavobacterium collinsii* strain CECT 7796 | GCA_902804485.1 | Rainbow trout (*O. mykiss*) | Spain | 2008 |
|  | *Flavobacterium columnare* strain ATCC 23463 | [GCA_002530675.1](https://www.ncbi.nlm.nih.gov/assembly/GCA_002530675.1) | Chinook salmon (*Oncorhynchus tshawytscha*) | USA | Unk |
|  | *Flavobacterium covae* strain 94-081* | CP013992.1 | Channel catfish (*Ictalurus punctatus*) | USA | 1994 |
|  | *Flavobacterium davisii* strain 90-106 | CP067378.1 | Channel catfish (*I. punctatus*) | USA | 1990 |
|  | *Flavobacterium erciyesense* strain F-328 | [GCA_018069925.1](https://www.ncbi.nlm.nih.gov/data-hub/genome/GCA_018069925.1/) | Rainbow trout (*O. mykiss*) | Turkey | 2017 |
|  | *Flavobacterium frigidimaris* strain DSM 15937 | GCA_002217275.1 | Sea water | Antarctica | 2003 |
|  | *Flavobacterium hibernum* strain ATCC 51468 | [GCA_002217315.1](https://www.ncbi.nlm.nih.gov/data-hub/genome/GCA_002217315.1/) | Lake | Antarctica | Unk |
|  | *Flavobacterium hydatis* strain ATCC 29551 | [GCA_002217335.1](https://www.ncbi.nlm.nih.gov/data-hub/genome/GCA_002217335.1/) | Hatchery | USA | 1974 |
|  | *Flavobacterium ichthyis* strain NST-5 | [GCA_009915155.1](https://www.ncbi.nlm.nih.gov/data-hub/genome/GCA_009915155.1/) | Water - fish pond | Taiwan | 2015 |
|  | *Flavobacterium indicum* strain GPTSA100-9 | HE774682.1 | Warmspring water | India | Unk |
|  | *Flavobacterium johnsoniae* strain ATCC 17061 | CP000685.1 | Soil | UK | Unk |
|  | *Flavobacterium kayseriense* strain F-47 | [GCA_014305095.1](https://www.ncbi.nlm.nih.gov/data-hub/genome/GCA_014305095.1/) | Rainbow trout (*O. mykiss*) | Unk | 2014 |
|  | *Flavobacterium limicola* strain DSM 15094 | [GCA_003634755.1](https://www.ncbi.nlm.nih.gov/data-hub/genome/GCA_003634755.1/) | River sediment | Japan | Unk |
|  | **Name** | **Accession** | **Source** | **Country** | **Collection date** |
|  | *Flavobacterium muglaense* strain F-60 | [GCA_014305155.1](https://www.ncbi.nlm.nih.gov/data-hub/genome/GCA_014305155.1/) | Rainbow trout (*O. mykiss*) | Turkey | 2013 |
|  | *Flavobacterium oncorhynchi* strain CCUG 59446 | [GCA_002217355.1](https://www.ncbi.nlm.nih.gov/data-hub/genome/GCA_002217355.1/) | Rainbow trout (*O. mykiss*) | Spain | 2008 |
|  | *Flavobacterium oreochromis* strain Costa Rica 04-02-TN | CP067377.1 | tilapia (*Oreochromis* sp.) | Costa Rica | 2004 |
|  | *Flavobacterium piscinae* strain ICH-30 | [GCA_004122145.1](https://www.ncbi.nlm.nih.gov/data-hub/genome/GCA_004122145.1/) | Water - fish pond | Taiwan | 2017 |
|  | *Flavobacterium piscis* strain CCUG 60099 | [GCA_001686925.1](https://www.ncbi.nlm.nih.gov/data-hub/genome/GCA_001686925.1/) | Rainbow trout (*O. mykiss*) | Madrid | 2009 |
|  | *Flavobacterium plurextorum* strain CCUG 60112 | [GCA_002217395.1](https://www.ncbi.nlm.nih.gov/data-hub/genome/GCA_002217395.1/) | Rainbow trout eggs (*O. mykiss*) | Spain | 2008 |
|  | *Flavobacterium proteolyticum* strain 1Y8A | [GCA_015223105.1](https://www.ncbi.nlm.nih.gov/data-hub/genome/GCA_015223105.1/) | Aquaculture water | China | 2019 |
|  | *Flavobacterium psychrolimnae* strain LMG 22018 | [GCA_003312425.1](https://www.ncbi.nlm.nih.gov/data-hub/genome/GCA_003312425.1/) | Antarctic lake | South Korea | 2018 |
|  | *Flavobacterium psychrophilum* ATCC 49418 | [GCA_002217405.1](https://www.ncbi.nlm.nih.gov/data-hub/genome/GCA_002217405.1/) | Coho salmon (*Oncorhynchus kisutch*) | USA | Unk |
|  | *Flavobacterium saccharophilum* strain DSM 1811 | [GCA_900142735.1](https://www.ncbi.nlm.nih.gov/data-hub/genome/GCA_900142735.1/) | Silt-water interface | UK | Unk |
|  | *Flavobacterium salmonis* strain CIP 111411 | [GCA_903819435.1](https://www.ncbi.nlm.nih.gov/data-hub/genome/GCA_903819435.1/) | Atlantic salmon (*S.* *salar*) | Chile | Unk |
|  | *Flavobacterium succinicans* strain DSM 4002 | [GCA_900114945.1](https://www.ncbi.nlm.nih.gov/data-hub/genome/GCA_900114945.1/) | Chinook salmon (*O. tshawytscha*) | USA | Unk |
|  | *Flavobacterium suncheonense* strain GH29-5 | [GCA_000769835.1](https://www.ncbi.nlm.nih.gov/data-hub/genome/GCA_000769835.1/) | Soil | South Korea | 2005 |
|  | *Flavobacterium tructae* strain CCUG 60100 | [GCA_002217475.1](https://www.ncbi.nlm.nih.gov/data-hub/genome/GCA_002217475.1/) | Fish farm | Spain | 2008 |
|  | *Flavobacterium turcicum* strain F-339 | GCA_011365745.1 | Rainbow trout (*O. mykiss*) | Turkey | 2017 |
| **Animal associated** | *Flavobacterium antarcticum* DSM 19726 | [GCA_000419685.1](https://www.ncbi.nlm.nih.gov/data-hub/genome/GCA_000419685.1/) | Soil sample of a penguin habitat | Antarctica | Unk |
|  | *Flavobacterium crassostreae* strain LPB0076 | CP017688.1 | Pacific oyster (*Crassostrea* *gigas*) | South Korea | Unk |
|  | *Flavobacterium cutihirudinis* strain DSM 25795 | [GCA_003385895.1](https://www.ncbi.nlm.nih.gov/data-hub/genome/GCA_003385895.1/) | Medical leech (*Hirudo* *verbana*) | Germany |  |
|  | *Flavobacterium faecale* strain WV33 | CP020918.1 | Penguin feces | Antarctica | 2011 |
|  | *Flavobacterium kingsejongi* strain WV39 | CP020919.1 | Feces of Antarctic penguins | Antarctica | 2011 |
|  | *Flavobacterium macacae* strain YIM 102600 | [GCA_003865365.1](https://www.ncbi.nlm.nih.gov/data-hub/genome/GCA_003865365.1/) | Macaque feces | China | 2016 |
|  | *Flavobacterium macrobrachii* strain an-8 | [GCA_014486675.1](https://www.ncbi.nlm.nih.gov/data-hub/genome/GCA_014486675.1/) | Shrimp pond | Taiwan | 2013 |
|  | *Flavobacterium profundi* strain TP390 | [GCA_006491645.1](https://www.ncbi.nlm.nih.gov/data-hub/genome/GCA_006491645.1/) | Marine sponge | Pacific Ocean | 2014 |
|  | *Flavobacterium segetis* strain DSM 19741 | [GCA_900129575.1](https://www.ncbi.nlm.nih.gov/data-hub/genome/GCA_900129575.1/) | Soil sample of a penguin habitat | Antarctica | Unk |
|  | *Flavobacterium tangerinum* strain YIM 102701-2 | [GCA_003865405.1](https://www.ncbi.nlm.nih.gov/data-hub/genome/GCA_003865405.1/) | Gibbon feces | China | 2016 |
|  | *Flavobacterium viscosum* strain YIM 102796 | GCA_003858535.1 | Primate feces | China | 2016 |
| **Environmental** | *Flavobacterium aciduliphilum* strain DSM 25663 | GCA_003268855.1 | Artificial lake | South Korea | Unk |
|  | *Flavobacterium ajazii* strain SSM4.2 | [GCA_010614725.1](https://www.ncbi.nlm.nih.gov/data-hub/genome/GCA_010614725.1/) | Seaweed | China | 2017 |
|  | *Flavobacterium akiainvivens* strain IK-1 | [GCA_001278115.1](https://www.ncbi.nlm.nih.gov/data-hub/genome/GCA_001278115.1/) | Wood (*Wikstroemia oahuensis*) | Hawaii | 2011 |
|  | *Flavobacterium album* strain HYN0059 | CP029186.1 | Freshwater | South Korea | 2016 |
|  | *Flavobacterium alkalisoli* strain XS-5 | CP042831.1 | Saline soil | China | 2018 |
|  | *Flavobacterium alvei* strain HR-AY | GCA_002920895.1 | Freshwater | South Korea | 2017 |
|  | *Flavobacterium amnicola* strain LLJ-11 | [GCA_004122165.1](https://www.ncbi.nlm.nih.gov/data-hub/genome/GCA_004122165.1/) | water | Taiwan | 2017 |
|  | *Flavobacterium amniphilum* strain KYPY10 | GCA_023634845.1 | Activated sludge | Taiwan | 2020 |
|  | *Flavobacterium aquariorum* IMCC3 4762 | GCA_003254745.1 | Freshwater | South Korea | 2016 |
|  | *Flavobacterium aquicola* strain DSM 100880 | GCA_003385115.1 | River water | Japan | 2013 |
|  | *Flavobacterium aurantiibacter* strain TH167 | GCA_002251775.1 | Cyanobacterial aggregates | China | 2016 |
|  | *Flavobacterium baculatum* strain SNL9 | [GCA_008629655.1](https://www.ncbi.nlm.nih.gov/data-hub/genome/GCA_008629655.1/) | Water | South Korea | 2018 |
|  | *Flavobacterium buctense* strain B7* | GCA_014486695.1 | Freshwater | South Korea | 2017 |
|  | *Flavobacterium caeni* strain CGMCC 1.7031 | GCA_900101895.1 | Sludge | Unk | Unk |
|  | *Flavobacterium caseinilyticum* strain AT-3-2 | GCA_004349085.1 | Artic soil | Svalbard | 2016 |
|  | **Name** | **Accession** | **Source** | **Country** | **Collection date** |
|  | *Flavobacterium cauense* strain R2A-7 | GCA_000498475.1 | Lake | China | Unk |
|  | *Flavobacterium celericrescens* strain TWA-26 | [GCA_011392075.1](https://www.ncbi.nlm.nih.gov/data-hub/genome/GCA_011392075.1/) | Water | Taiwan | 2018 |
|  | *Flavobacterium cellulosilyticum* strain AR-3-4 | GCA_004349355.1 | Artic soil | Svalbard | 2016 |
|  | *Flavobacterium cerinum* strain 1E403 | [GCA_004028155.1](https://www.ncbi.nlm.nih.gov/data-hub/genome/GCA_004028155.1/) | Soil | Norway | 2017 |
|  | *Flavobacterium cheniae* strain CGMCC 1.6844 | [GCA_007830415.1](https://www.ncbi.nlm.nih.gov/data-hub/genome/GCA_007830415.1/) | Sediment of the eutrophicated Guanting Reservoir | China | Unk |
|  | *Flavobacterium cheongpyeongense* strain IMCC 34759 | [GCA_003202435.1](https://www.ncbi.nlm.nih.gov/data-hub/genome/GCA_003202435.1/) | Water | Unk | 2017 |
|  | *Flavobacterium chungangense* LMG 26729* | GCA_000735715.2 | Lake | South Korea | 2009 |
|  | *Flavobacterium chungbukense* strain CS100 | [GCA_020805785.1](https://www.ncbi.nlm.nih.gov/data-hub/genome/GCA_020805785.1/) | Soil | South Korea | 2010 |
|  | *Flavobacterium circumlabens* strain P5626 | [GCA_004345565.1](https://www.ncbi.nlm.nih.gov/assembly/GCA_004345565.1) | Sediment from upper reaches of temporary stream | Antarctica | 2014 |
|  | *Flavobacterium commune* strain PK15 | CP017774.1 | Freshwater | South Korea | 2012 |
|  | *Flavobacterium coralii* strain D11R37 | [GCA_019891545.1](https://www.ncbi.nlm.nih.gov/data-hub/genome/GCA_019891545.1/) | Seawater | China | 2021 |
|  | *Flavobacterium croceum* strain DSM 17960 | GCA_002917885.1 | Activated sludge | South Korea | 2005 |
|  | *Flavobacterium crocinum* strain HYN0056 | CP029255.1 | Freshwater | South Korea | 2016 |
|  | *Flavobacterium cucumis* strain DSM 18830 | GCA_900148835.1 | Greenhouse soil cultivated with cucumber | South Korea | Unk |
|  | *Flavobacterium cupreum* strain CCM 8825 | [GCA_003996965.1](https://www.ncbi.nlm.nih.gov/data-hub/genome/GCA_003996965.1/) | Regolith | Antarctica | 2008 |
|  | *Flavobacterium daemonense* strain JCM 19455 | [GCA_007341385.1](https://www.ncbi.nlm.nih.gov/data-hub/genome/GCA_007341385.1/) | Soil | South Korea | 2015 |
|  | *Flavobacterium dankookense* strain DSM 25687 | GCA_004362665.1 | Freshwater lake | South Korea | Unk |
|  | *Flavobacterium defluvii* strain DSM 17963 | GCA_900129555.1 | Activated sludge | South Korea | Unk |
|  | *Flavobacterium degerlachei* strain DSM 15718 | [GCA_900106645.1](https://www.ncbi.nlm.nih.gov/data-hub/genome/GCA_900106645.1/) | Microbial mat | Antarctica | Unk |
|  | *Flavobacterium endoglycinae* strain BB8 | CP071448.1 | Soybean | South Korea | 2020 |
|  | *Flavobacterium endophyticum* strain DSM 29537 | [GCA_003634455.1](https://www.ncbi.nlm.nih.gov/data-hub/genome/GCA_003634455.1/) | Maize root | China | Unk |
|  | *Flavobacterium enshiense* strain DK69 | [GCA_000498495.1](https://www.ncbi.nlm.nih.gov/data-hub/genome/GCA_000498495.1/) | Soil | China | Unk |
|  | *Flavobacterium filum* strain DSM 17961 | GCA_000425465.1 | Activated sludge | South Korea | Unk |
|  | *Flavobacterium flevense* strain DSM 1076 | [GCA_900142775.1](https://www.ncbi.nlm.nih.gov/data-hub/genome/GCA_900142775.1/) | Lake water | Netherlands | Unk |
|  | *Flavobacterium fluviale* strain HYN0086 | CP030261.1 | Fresh water | South Korea | 2017 |
|  | *Flavobacterium fluviatile* strain TAPY14 | GCA_010645065.1 | Freshwater creek | Taiwan | 2016 |
|  | *Flavobacterium fluvii* strain DSM 19978 | [GCA_900129545.1](https://www.ncbi.nlm.nih.gov/data-hub/genome/GCA_900129545.1/) | Stream sediment | South Korea | Unk |
|  | *Flavobacterium fontis* strain DSM 25660 | GCA_900129405.1 | Freshwater pond | South Korea | 2011 |
|  | *Flavobacterium franklandianum* strain LB3P56 | GCA_007097365.1 | Glacier | China | 2016 |
|  | *Flavobacterium frigoris* strain DSM 15719 | GCA_900111075.1 | Microbial mat | Antarctica | Unk |
|  | *Flavobacterium fryxellicola* strain DSM 16209 | [GCA_900143245.1](https://www.ncbi.nlm.nih.gov/data-hub/genome/GCA_900143245.1/) | Microbial mat | Antarctica | Unk |
|  | *Flavobacterium gawalongense* strain GSR18* | GCA_007097285.1 | Glacier | China | 2016 |
|  | *Flavobacterium gelidilacus* strain DSM 15343 | [GCA_000422685.1](https://www.ncbi.nlm.nih.gov/data-hub/genome/GCA_000422685.1/) | Microbial mat | Antarctica | Unk |
|  | *Flavobacterium gilvum* strain EM1308 | CP017479.1 | Streamwater | South Korea | 2013 |
|  | *Flavobacterium ginsenosidimutans* strain THG 01 | [GCA_003254625.1](https://www.ncbi.nlm.nih.gov/data-hub/genome/GCA_003254625.1/) | Soil | South Korea | 2011 |
|  | *Flavobacterium glaciei* strain CGMCC 1.5380 | [GCA_007994155.1](https://www.ncbi.nlm.nih.gov/data-hub/genome/GCA_007994155.1/) | Glacier sediment | China | 2006 |
|  | *Flavobacterium glycines* strain Gm-149 | [GCA_900100165.1](https://www.ncbi.nlm.nih.gov/data-hub/genome/GCA_900100165.1/) | Rhizosphere | India | Unk |
|  | *Flavobacterium gossypii* strain DSM 100397 | GCA_014138495.1 | Cotton (*Gossypium* *hirsutum*) | USA | Unk |
|  | *Flavobacterium granuli* strain DSM 17797 | GCA_003003155.1 | Granule sludge | South Korea | Unk |
|  | *Flavobacterium haoranii* strain DSM 22807 | GCA_900142055.1 | Activated sludge | China | Unk |
|  | *Flavobacterium hercynium* strain DSM 18292 | [GCA_002217285.1](https://www.ncbi.nlm.nih.gov/data-hub/genome/GCA_002217285.1/) | Stream water | Germany | Unk |
|  | *Flavobacterium hibisci* strain THG-HG1.4 | GCA_020026925.1 | Flower rhizosphere | South Korea | 2016 |
|  | **Name** | **Accession** | **Source** | **Country** | **Collection date** |
|  | *Flavobacterium hiemivividum* strain TSA-D2 | GCA_004349145.1 | Arctic soil | Svalbard | 2016 |
|  | *Flavobacterium hydrocarbonoxydans* strain GA093 | [GCA_009789235.1](https://www.ncbi.nlm.nih.gov/data-hub/genome/GCA_009789235.1/) | Polluted soil | South Korea | 2018 |
|  | *Flavobacterium hydrophilum* strain IMCC 34758 | [GCA_003202405.1](https://www.ncbi.nlm.nih.gov/data-hub/genome/GCA_003202405.1/) | Water | South Korea | 2017 |
|  | *Flavobacterium jejuense* strain EC11 | GCA_006491595.2 | Brown alga | South Korea | 2013 |
|  | *Flavobacterium jumunjinense* strain HME7102 | CP091285.1 | Sihwaho lake | South Korea | 2020 |
|  | *Flavobacterium lacus* strain CGMCC 1.12504 | [GCA_003268815.1](https://www.ncbi.nlm.nih.gov/data-hub/genome/GCA_003268815.1/) | High-altitude lake | China | Unk |
|  | *Flavobacterium laiguense* strain LB2P30 | [GCA_003097655.1](https://www.ncbi.nlm.nih.gov/data-hub/genome/GCA_003097655.1/) | Glacier ice | China | 2016 |
|  | *Flavobacterium limi* strain CGMCC 1.16060 | GCA_014644395.1 | Forest mud | South Korea | Unk |
|  | *Flavobacterium lindanitolerans* strain DSM 21886 | [GCA_003663835.1](https://www.ncbi.nlm.nih.gov/data-hub/genome/GCA_003663835.1/) | Polluted soil | India | Unk |
|  | *Flavobacterium litorale* strain WSW3-B6 | CP080429.1 | Seaweed | South Korea | 2020 |
|  | *Flavobacterium lotistagni* strain CYK-4 | [GCA_011392125.1](https://www.ncbi.nlm.nih.gov/data-hub/genome/GCA_011392125.1/) | Water | Taiwan | 2018 |
|  | *Flavobacterium luteum* strain NBRC 112527 | [GCA_008806775.1](https://www.ncbi.nlm.nih.gov/data-hub/genome/GCA_008806775.1/) | Glacier ice | Unk | Unk |
|  | *Flavobacterium lutivivi* strain CGMCC 1.15347 | GCA_014642275.1 | Activated sludge | Unk | Unk |
|  | *Flavobacterium magnum* strain HYN0048 | CP028811.1 | Fresh water | South Korea | 2016 |
|  | *Flavobacterium microcysteis* strain MaA-Y11 | [GCA_006385255.1](https://www.ncbi.nlm.nih.gov/data-hub/genome/GCA_006385255.1/) | River | South Korea | 2013 |
|  | *Flavobacterium micromati* strain DSM 17659 | [GCA_900129585.1](https://www.ncbi.nlm.nih.gov/data-hub/genome/GCA_900129585.1/) | Microbial mat | Antarctica | Unk |
|  | *Flavobacterium nackdongense* strain GS13 | CP037933.1 | Water | South Korea | 2018 |
|  | *Flavobacterium nitrogenifigens* strain DSM 29982 | GCA_900182675.1 | Switchgrass (*Panicum* *virgatum*) | USA | 1998 |
|  | *Flavobacterium noncentrifugens* strain CGMCC 1.10076 | [GCA_900100375.1](https://www.ncbi.nlm.nih.gov/data-hub/genome/GCA_900100375.1/) | Glacier meltwater | Unk | Unk |
|  | *Flavobacterium notoginsengisoli* strain S00128 | [GCA_014207905.1](https://www.ncbi.nlm.nih.gov/data-hub/genome/GCA_014207905.1/) | Rhizosphere of *Panax* *notoginseng* | USA | Unk |
|  | *Flavobacterium orientale* strain CGMCC 1.12506 | [GCA_014638005.1](https://www.ncbi.nlm.nih.gov/data-hub/genome/GCA_014638005.1/) | Lake water | China | Unk |
|  | *Flavobacterium pallidum* strain HYN0049 | CP029187.1 | Freshwater | South Korea | 2016 |
|  | *Flavobacterium panici* strain PXU-55 | GCA_903819335.1 | Switchgrass (*Panicum* *virgatum*) | USA | 2012 |
|  | *Flavobacterium pectinovorum* strain ATCC 19366 | [GCA_002217365.1](https://www.ncbi.nlm.nih.gov/data-hub/genome/GCA_002217365.1/) | Soil | UK | Unk |
|  | *Flavobacterium phycosphaerae* strain MK012 | GCA_010119235.1 | Freshwater | South Korea | 2019 |
|  | *Flavobacterium pokkalii* strain L1I52 | [GCA_014596575.1](https://www.ncbi.nlm.nih.gov/data-hub/genome/GCA_014596575.1/) | Rhizosphere | India | 2014 |
|  | *Flavobacterium psychroterrae* strain CCM 8827 | [GCA_018380615.1](https://www.ncbi.nlm.nih.gov/data-hub/genome/GCA_018380615.1/) | Stone fissure | Antarctica | 2009 |
|  | *Flavobacterium psychrotolerans* strain RB1R5 | [GCA_003097635.1](https://www.ncbi.nlm.nih.gov/data-hub/genome/GCA_003097635.1/) | Glacier ice | China | 2016 |
|  | *Flavobacterium quisquiliarum* strain EA-12 | GCA_019308285.1 | Activated sludge | China | 2017 |
|  | *Flavobacterium rakeshii* strain JCM 17928 | [GCA_009741375.1](https://www.ncbi.nlm.nih.gov/data-hub/genome/GCA_009741375.1/) | Sediment | India | 2012 |
|  | *Flavobacterium ranwuense* strain LB2P22 | GCA_004349315.1 | Glacier | China | 2016 |
|  | *Flavobacterium reichenbachii* strain DSM 21791 | [GCA_002217435.1](https://www.ncbi.nlm.nih.gov/data-hub/genome/GCA_002217435.1/) | Water | Germany | Unk |
|  | *Flavobacterium resistens* strain DSM 19382 | [GCA_900182645.1](https://www.ncbi.nlm.nih.gov/data-hub/genome/GCA_900182645.1/) | Stream sediment | South Korea | Unk |
|  | *Flavobacterium restrictum* strain LB1R34 | GCA_007097245.1 | Glacier | China | 2016 |
|  | *Flavobacterium rhamnosiphilum* strain LB3P52 | GCA_004349195.1 | Glacier | China | 2016 |
|  | *Flavobacterium salilacus* strain SaA2.12 | [GCA_004634195.1](https://www.ncbi.nlm.nih.gov/data-hub/genome/GCA_004634195.1/) | Lake water | China | 2017 |
|  | *Flavobacterium saliperosum* strain CGMCC 1.3801 | [GCA_900100625.1](https://www.ncbi.nlm.nih.gov/data-hub/genome/GCA_900100625.1/) | Lake sediment | China | Unk |
|  | *Flavobacterium sandaracinum* strain LB-D12 | GCA_004349135.1 | Arctic soil | Svalbard | 2016 |
|  | *Flavobacterium sangjuense* strain GS03 | CP038810.1 | Sediment | South Korea | 2018 |
|  | *Flavobacterium sediminilitoris* strain YSM-43 | CP090145.1 | Tidal flat | South Korea | Unk |
|  | *Flavobacterium sediminis* strain MEBiC07310 | CP029463.1 | Tidal sediment | South Korea | 2010 |
|  | *Flavobacterium seoulense* strain EM1321 | [GCA_000695795.1](https://www.ncbi.nlm.nih.gov/data-hub/genome/GCA_000695795.1/) | Stream water | South Korea | 2013 |
|  | **Name** | **Accession** | **Source** | **Country** | **Collection date** |
|  | *Flavobacterium sharifuzzamanii* strain A7.6 | [GCA_003254585.1](https://www.ncbi.nlm.nih.gov/data-hub/genome/GCA_003254585.1/) | Marine sediment | China | 2017 |
|  | *Flavobacterium silvaticum* strain SE-s28 | GCA_012911565.1 | Forest soil | South Korea | 2016 |
|  | *Flavobacterium silvisoli* strain RD-2-33 | [GCA_004329815.1](https://www.ncbi.nlm.nih.gov/data-hub/genome/GCA_004329815.1/) | Soil | South Korea | 2018 |
|  | *Flavobacterium sinopsychrotolerans* strain CGMCC 1.8704 | GCA_900110375.1 | Glacier | China | Unk |
|  | *Flavobacterium soli* strain DSM 19725 | [GCA_000422705.1](https://www.ncbi.nlm.nih.gov/data-hub/genome/GCA_000422705.1/) | Soil | South Korea | Unk |
|  | *Flavobacterium solisilvae* strain SE-s27 | GCA_012911605.1 | Forest soil | South Korea | 2016 |
|  | *Flavobacterium soyae* strain SCIV07 | [GCA_021245985.1](https://www.ncbi.nlm.nih.gov/data-hub/genome/GCA_021245985.1/) | Rhizosphere soil | China | 2019 |
|  | *Flavobacterium soyangense* strain CGMCC 1.13493 | [GCA_015277675.1](https://www.ncbi.nlm.nih.gov/data-hub/genome/GCA_015277675.1/) | Freshwater lake | South Korea | Unk |
|  | *Flavobacterium stagni* strain WWJ-16 | [GCA_004122105.1](https://www.ncbi.nlm.nih.gov/data-hub/genome/GCA_004122105.1/) | Water | Taiwan | 2016 |
|  | *Flavobacterium suaedae* strain CGMCC 1.15461 | [GCA_014642915.1](https://www.ncbi.nlm.nih.gov/data-hub/genome/GCA_014642915.1/) | Root of *Suaeda corniculata* | China | Unk |
|  | *Flavobacterium subsaxonicum* strain WB 4.1-42 | [GCA_000769935.1](https://www.ncbi.nlm.nih.gov/data-hub/genome/GCA_000769935.1/) | Water | Germany | 2007 |
|  | *Flavobacterium sufflavum* strain BBQ-12 | [GCA_004016525.1](https://www.ncbi.nlm.nih.gov/data-hub/genome/GCA_004016525.1/) | Water | Taiwan | 2016 |
|  | *Flavobacterium supellecticarium* strain CC-CTC003 | [GCA_004801375.1](https://www.ncbi.nlm.nih.gov/data-hub/genome/GCA_004801375.1/) | Synthetic wooden board | Taiwan | Unk |
|  | *Flavobacterium swingsii* strain DSM 21789 | [GCA_900111965.1](https://www.ncbi.nlm.nih.gov/data-hub/genome/GCA_900111965.1/) | Hardwater creek | Germany | Unk |
|  | *Flavobacterium taihuense* strain NAS39 | [GCA_019351435.1](https://www.ncbi.nlm.nih.gov/data-hub/genome/GCA_019351435.1/) | Lake sediment | China | 2018 |
|  | *Flavobacterium tegetincola* strain DSM 22377 | GCA_000425485.1 | Cyanobacterial mat | Antarctica | Unk |
|  | *Flavobacterium terrae* strain DSM 18829 | [GCA_900142035.1](https://www.ncbi.nlm.nih.gov/data-hub/genome/GCA_900142035.1/) | Greenhouse soil cultivated with lettuce | South Korea | Unk |
|  | *Flavobacterium terrigena* strain DSM 17934A01.1 | [GCA_900108955.1](https://www.ncbi.nlm.nih.gov/data-hub/genome/GCA_900108955.1/) | Soil | South Korea | Unk |
|  | *Flavobacterium tiangeerense* strain CGMCC 1.6847 | GCA_007830355.1 | Glacier | China | Unk |
|  | *Flavobacterium tibetense* strain YH5 | [GCA_003293845.1](https://www.ncbi.nlm.nih.gov/data-hub/genome/GCA_003293845.1/) | Wetland | China | 2018 |
|  | *Flavobacterium tistrianum* strain GB 56.1 | [GCA_003254545.1](https://www.ncbi.nlm.nih.gov/assembly/GCA_003254545.1) | Soil | Thailand | 2016 |
|  | *Flavobacterium tyrosinilyticum* strain KCTC 42726 | [GCA_023656565.1](https://www.ncbi.nlm.nih.gov/data-hub/genome/GCA_023656565.1/) | Rhizosphere of strawberry plant | South Korea | 2022 |
|  | *Flavobacterium ummariense* strain DS-12 | [GCA_900115115.1](https://www.ncbi.nlm.nih.gov/data-hub/genome/GCA_900115115.1/) | Soil | India | Unk |
|  | *Flavobacterium urocaniciphilum* strain DSM 27078 | [GCA_900110615.1](https://www.ncbi.nlm.nih.gov/data-hub/genome/GCA_900110615.1/) | Wastewater sludge | Japan | Unk |
|  | *Flavobacterium urumqiense* strain CGMCC 1.9230 | GCA_900108015.1 | Glacier | China | Unk |
|  | *Flavobacterium ustbae* strain T13 | [GCA_003946915.1](https://www.ncbi.nlm.nih.gov/assembly/GCA_003946915.1) | Rhizosphere soil | China | 2017 |
|  | *Flavobacterium weaverense* strain DSM 19727 | [GCA_003688495.1](https://www.ncbi.nlm.nih.gov/data-hub/genome/GCA_003688495.1/) | Soil | Antarctica | Unk |
|  | *Flavobacterium xanthum* strain DSM 3661 | [GCA_900142695.1](https://www.ncbi.nlm.nih.gov/data-hub/genome/GCA_900142695.1/) | Soil | Antarctica | Unk |
|  | *Flavobacterium zepuense* strain ZT4R6 | GCA_007097145.1 | Glacier | China | 2016 |
|  | *Flavobacterium zhairuonense* strain A5.7 | GCA_004352915.1 | Soil | South Korea | 2015 |

**STable 2**: *Chryseobacterium* strains with publicly available whole genomes used in phylogenetic analysis. Reference genomes used when type-strain genomes were not available are indicated with an asterisk. Host association (fish or other animal) may not reflect the isolation source of the type-strain but subsequent published isolations.

|  | **Name** | **Accession** | **Source** | **Country** | **Collection date** |
| --- | --- | --- | --- | --- | --- |
| **Fish associated** | *Chryseobacterium aahli* strain cx-42* | [GCA_022699665.1](https://www.ncbi.nlm.nih.gov/data-hub/genome/GCA_022699665.1/) | Lake trout (*Salvelinus namaycush*) | USA | 2014 |
|  | *Chryseobacterium aquaticum* strain KCTC 12483 | GCA_001420285.1 | Water reservoir | South Korea | 2007 |
|  | *Chryseobacterium aurantiacum* strain F30 | [GCA_003020585.1](https://www.ncbi.nlm.nih.gov/data-hub/genome/GCA_003020585.1/) | Water- fish farm with diseased Murray cod (*Maccullochella peelii*) | China | 2017 |
|  | *Chryseobacterium balustinum* strain DSM 16775 | GCA_900168205.1 | Fish blood | Switzerland | 1978 |
|  | *Chryseobacterium bernardetii* strain G0229 | CP033932.1 | Human (*Homo sapiens*) | UK | 1982 |
|  | *Chryseobacterium chaponense* strain DSM 23145 | GCA_900156725.1 | Atlantic salmon (*Salmo salar*) | Chile | 2009 |
|  | *Chryseobacterium cucumeris* strain GSE06 | GCA_001593385.1 | Cucumber (*Cucumis sativus*) | South Korea | 2002 |
|  | *Chryseobacterium gleum* strain NCTC 11432 | LR134289.1 | Human (*Homo* *sapiens*) | UK | 1982 |
|  | *Chryseobacterium haifense* strain DSM 19056 | GCA_000735695.2 | Raw milk | Israel | 2004 |
|  | *Chryseobacterium indologenes* strain NCTC 10796 | GCA_900460995.1 | Human | Unk | 1958 |
|  | *Chryseobacterium indoltheticum* strain ATCC 27950 | CP033929.1 | Marine mud | Unk | Unk |
|  | *Chryseobacterium joostei* strain DSM 16927 | CP033926.1 | Raw milk | South Africa | 1981 |
|  | *Chryseobacterium oncorhynchi* strain 701B-08 | GCA_002899895.2 | Rainbow trout (*Oncorhynchus mykiss*) | Spain | 2017 |
|  | *Chryseobacterium piscicola* strain DSM 21068 | GCA_002943675.1 | Atlantic salmon (*Salmo salar*) | Chile | Unk |
|  | *Chryseobacterium piscium* strain CCUG 51923 | GCA_003385415.1 | *Merluccius capensis* | South Africa | 1996 |
|  | *Chryseobacterium scophthalmum* strain DSM 16779 | GCA_900143185.1 | Turbot (*Scophthalmus maximus*) | UK | Unk |
|  | *Chryseobacterium shigense* strain DSM 17126 | GCA_002943655.1 | Lactic acid beverage | Japan | 2001 |
|  | *Chryseobacterium viscerum* strain 687B-08 | GCA_002899945.2 | Rainbow trout (*Oncorhynchus mykiss*) | Spain | 2008 |
|  | *Chryseobacterium vrystaatense* strain LMG 22846 | GCA_000737765.1 | Raw chicken | South Africa | Unk |
| **Animal and animal products** | *Chryseobacterium arthrosphaerae* CC-VM-7 | GCA_001684965.1 | Pill millipede | India | 2010 |
|  | *Chryseobacterium capnotolerans* isolate DH-B6 | [CP065589.1](https://www.ncbi.nlm.nih.gov/nuccore/CP065589.1/) | Pork sausage | Germany | 2019 |
|  | *Chryseobacterium carnipullorum* strain NCTC 13533 | GCA_900446825.1 | Raw chicken | South Africa | 2002 |
|  | *Chryseobacterium carnis* strain G0081* | [CP034159.1](https://www.ncbi.nlm.nih.gov/nuccore/CP034159.1/) | Beef | UK | 1973 |
|  | *Chryseobacterium culicis* strain DSM 23031 | GCA_900108365.1 | Mosquito (*Culex* *quinquefasciatus*) | India | Unk |
|  | *Chryseobacterium echinoideorum* strain CC-CZW010 | GCA_007474535.1 | Sea urchin | China | 2015 |
|  | *Chryseobacterium faecale* strain F4 | [CP087583.1](https://www.ncbi.nlm.nih.gov/nuccore/CP087583.1/) | Camel feces | South Korea | 2020 |
|  | *Chryseobacterium gallinarum* strain DSM 27622 | GCA_001021975.1 | Chicken | Germany | 2014 |
|  | *Chryseobacterium lactis* strain KC_1864 | GCA_003815875.1 | Milk bottle rinse | UK | Unk |
|  | *Chryseobacterium mulctrae* strain CA10 | [GCA_006175945.1](https://www.ncbi.nlm.nih.gov/data-hub/genome/GCA_006175945.1/) | Raw milk | South Korea | 2017 |
|  | *Chryseobacterium nakagawai* strain G0041 | CP033923.1 | Clinical isolate (*Homo sapiens*) | UK | Unk |
|  | *Chryseobacterium oranimense* strain DSM 19055 | GCA_900129755.1 | Raw milk | Israel | Unk |
|  | *Chryseobacterium pennae* strain 1_F178 | GCA_003385515.1 | Poultry litter | South Africa | 2011 |
|  | *Chryseobacterium pennipullorum* strain 7_F195 | [GCA_003384725.1](https://www.ncbi.nlm.nih.gov/assembly/GCA_003384725.1) | Poultry litter | South Africa | 2011 |
|  | *Chryseobacterium schmidteae* strain Marseille-P9602 | GCA_903166575.1 | Planarian (*Schmidtea mediterranea*) | France | 2019 |
|  | **Name** | **Accession** | **Source** | **Country** | **Collection date** |
|  | *Chryseobacterium shandongense* strain G0239 | CP033914.1 | Cryoprecipitate | UK | 1982 |
|  | *Chryseobacterium takakiae* strain DSM 26898 | GCA_900129385.1 | Bryophyte (*Takakia lepidozioides*) | China | Unk |
|  | *Chryseobacterium taklimakanense* strain NCTC 13490 | GCA_900187185.1 | Soil | China | 2009 |
|  | *Chryseobacterium timonianum* strain G972 | GCA_900078205.2 | Clinical isolate (*Homo sapiens*) | France | Unk |
|  | *Chryseobacterium treverense* strain DSM 22251 | GCA_900114045.1 | Blood (*Homo sapiens*) | Germany | 1996 |
|  | *Chryseobacterium vaccae* strain CA7 | GCA_009602705.1 | Raw cow's milk | South Korea | 2017 |
| **Environmental** | *Chryseobacterium antibioticum* strain RP-3-3 | GCA_012927325.1 | Arctic soil | Canada | 2016 |
|  | *Chryseobacterium aquaeductus* strain CECT 9390 | [GCA_905175375.1](https://www.ncbi.nlm.nih.gov/data-hub/genome/GCA_905175375.1/) | Drinking water | Spain | 2017 |
|  | *Chryseobacterium arachidis* DSM 27619 | GCA_900129245.1 | Geocarposphere - peanuts (*Arachis hypogaea)* | USA | Unk |
|  | *Chryseobacterium artocarpi* strain UTM-3 | [GCA_001684975.1](https://www.ncbi.nlm.nih.gov/data-hub/genome/GCA_001684975.1/) | Rhizosphere | Malaysia | 2011 |
|  | *Chryseobacterium aureum* strain 17S1E7 | CP034661.1 | Freshwater | South Korea | 2017 |
|  | *Chryseobacterium binzhouense* strain LM2 | [GCA_007474545.1](https://www.ncbi.nlm.nih.gov/data-hub/genome/GCA_007474545.1/) | Activated sludge | China | 2018 |
|  | *Chryseobacterium camelliae* strain Dolsongi-HT1* | [CP022986.1](https://www.ncbi.nlm.nih.gov/nuccore/CP022986.1/) | Leaf (*Camellia sinensis*) | South Korea | 2016 |
|  | *Chryseobacterium candidae* strain JC507 | [GCA_004916905.1](https://www.ncbi.nlm.nih.gov/data-hub/genome/GCA_004916905.1/) | Yeast | India | 2017 |
|  | *Chryseobacterium caseinilyticum* strain GCR10 | [GCA_014837145.1](https://www.ncbi.nlm.nih.gov/data-hub/genome/GCA_014837145.1/) | Plant | South Korea | 2020 |
|  | *Chryseobacterium cheonjiense* strain RJ-7-14 | [GCA_012927265.1](https://www.ncbi.nlm.nih.gov/data-hub/genome/GCA_012927265.1/) | Soil | South Korea | 2019 |
|  | *Chryseobacterium contaminans* strain DSM 27621 | GCA_900142615.1 | Contaminant on rhizosphere sample | USA | 2013 |
|  | *Chryseobacterium daecheongense* strain DSM 15235 | GCA_004365465.1 | Sediment - sewage | South Korea | Unk |
|  | *Chryseobacterium daeguense* DSM 19388 | GCA_000430825.1 | Wastewater | South Korea | 2004 |
|  | *Chryseobacterium defluvii* strain DSM 14219 | GCA_003634775.1 | Wastewater | Germany | 1998 |
|  | *Chryseobacterium elymi* strain KCTC 22547 | GCA_003385495.1 | Rhizosphere - wild rye | South Korea | 2005 |
|  | *Chryseobacterium endalhagicum* strain L7 | [GCA_016745235.1](https://www.ncbi.nlm.nih.gov/data-hub/genome/GCA_016745235.1/) | Seed (*Alhagi sparsifolia*) | China | 2017 |
|  | *Chryseobacterium fistulae* strain CECT 9393 | [GCA_902729325.1](https://www.ncbi.nlm.nih.gov/data-hub/genome/GCA_902729325.1/) | Drinking water | Spain | 2017 |
|  | *Chryseobacterium flavum* strain KCTC 12877 | GCA_003385595.1 | Soil - polluted | China | 2007 |
|  | *Chryseobacterium formosense* strain DSM 17452 | GCA_900116415.1 | Rhizosphere of *Lactuca sativa* | Taiwan | Unk |
|  | *Chryseobacterium frigidisoli* strain DSM 26000 | GCA_900113805.1 | Glacier | Antarctica | Unk |
|  | *Chryseobacterium gambrini* strain DSM 18014 | GCA_900156825.1 | Steel surface of a beer-bottling plant | Germany | 2002 |
|  | *Chryseobacterium geocarposphaerae* strain DSM 27617 | GCA_002797535.1 | Geocarposphere of peanuts (*Arachis hypogaea*) | USA | Unk |
|  | *Chryseobacterium glaciei* strain IHBB 10212 | [CP015199.1](https://www.ncbi.nlm.nih.gov/nuccore/CP015199.1/) | Glacier | India | 2011 |
|  | *Chryseobacterium gwangjuense* strain THG-A18 | [GCA_021311115.1](https://www.ncbi.nlm.nih.gov/data-hub/genome/GCA_021311115.1/) | Soil | South Korea | 2021 |
|  | *Chryseobacterium hagamense* NBRC 105253 | [GCA_007991455.1](https://www.ncbi.nlm.nih.gov/data-hub/genome/GCA_007991455.1/) | Rhizosphere | South Korea | Unk |
|  | *Chryseobacterium hispalense* DSM 25574 | GCA_000708615.2 | Water - pond | Spain | Unk |
|  | *Chryseobacterium humi* strain DSM 21580 | GCA_900108025.1 | Soil - polluted | Portugal | Unk |
|  | *Chryseobacterium jejuense* strain DSM 19299 | GCA_900100075.1 | Soil | South Korea | 2005 |
|  | *Chryseobacterium koreense* strain DSM 25209 | GCA_014201785.1 | Fresh mineral water | South Korea | 2004 |
|  | *Chryseobacterium kwangjuense* strain KJ1R5 | GCA_001563495.1 | Pepper (*Capsicum annuum*) | South Korea | 2001 |
|  | *Chryseobacterium lacus* strain YLOS41 | [GCA_003336205.1](https://www.ncbi.nlm.nih.gov/data-hub/genome/GCA_003336205.1/) | Water - lake | China | 2017 |
|  | *Chryseobacterium lathyri* strain KCTC 22544 | GCA_003290185.1 | wild pea (*Lathyrus japonica*) | South Korea | 2005 |
|  | *Chryseobacterium luteum* strain DSM 18605 | GCA_000737785.1 | Phyllosphere of grasses | Germany | 2013 |
|  | *Chryseobacterium nepalense* strain AC3* | [CP096203.1](https://www.ncbi.nlm.nih.gov/nuccore/CP096203.1/) | Soil | China | 2020 |
|  | **Name** | **Accession** | **Source** | **Country** | **Collection date** |
|  | *Chryseobacterium oleae* strain DSM 25575 | GCA_900115055.1 | Rhizosphere of olive (*Olea europae*) | Spain | 2006 |
|  | *Chryseobacterium palustre* DSM 21579 | GCA_000422265.1 | Rhizosphere - polluted | Portugal | Unk |
|  | *Chryseobacterium panacisoli* strain Gsoil 183 | GCA_008274625.1 | Ginseng field | South Korea | 2006 |
|  | *Chryseobacterium phosphatilyticum* strain ISE14 | GCA_002899825.2 | Cucumber | South Korea | 2002 |
|  | *Chryseobacterium piperi* strain ATCC BAA-1782 | CP023049.2 | Sediment - freshwater creek | USA | 2006 |
|  | *Chryseobacterium polytrichastri* strain DSM 26899 | GCA_900142445.1 | Surface-disinfected moss (*Polytrichastrum formosum*) | China | 2012 |
|  | *Chryseobacterium populi* strain CF314 | GCA_000282115.1 | Eastern cottonwood (P*opulus deltoides*) | USA | Unk |
|  | *Chryseobacterium salipaludis* strain LZ-2022a* | GCA_022968825.1 | Sediment - salt marsh, wild ass sanctuary | India | 2018 |
|  | *Chryseobacterium salivictor* strain NBC 122 | CP037954.1 | Freshwater | South Korea | 2018 |
|  | *Chryseobacterium soldanellicola* strain DSM 17072 | GCA_900100115.1 | Root of *Calystegia soldanella* | South Korea | Unk |
|  | *Chryseobacterium soli* strain DSM 19298 | GCA_000737705.1 | Soil | South Korea | 2014 |
|  | *Chryseobacterium taeanense* strain DSM 17071  **Environmental** | GCA_900099685.1 | Root of*Elymus mollis* | South Korea | Unk |
|  | *Chryseobacterium taichungense* strain DSM 17453 | GCA_900109935.1 | Soil - polluted | Taiwan | Unk |
|  | *Chryseobacterium taihuense* strain CGMCC 1.10941 | GCA_900103755.1 | Decomposing algal scum | Unk | Unk |
|  | *Chryseobacterium taiwanense* strain TPW19* | GCA_000813825.1 | Waterfall | Malaysia | 2013 |
|  | *Chryseobacterium ureilyticum* strain DSM 18017 | GCA_900156735.1 | Steel surface of a beer-bottling plant | Germany | 2004 |
|  | *Chryseobacterium wanjuense* strain DSM 17724 | GCA_900111495.1 | Soil - cultivated with lettuce (*Lactuca sativa*) | South Korea | Unk |

**STable 3:** Isolates collected from diseased fish in the Western US identified as *Flavobacterium* species. The *gyrB* clade designation and coloration reflect genetic groupings in corresponding phylogenetic trees (Figure 1). The closest accepted species was determined by BLAST alignment of isolate 16S rRNA sequence fragments to a local database populated by type-strain 16S rRNA genes. Pairwise ID is the average percent identity over the alignment. Isolates in bold were used in antimicrobial testing.

| **Isolate name** | **Clade** | **Pairwise ID (%)** | **Closest species** | **Host** | **State** | **Date** |
| --- | --- | --- | --- | --- | --- | --- |
| CA 125 | **A** | 100 | *F. columnare* | Coho salmon (*Oncorhynchus* *kisutch*) | CA | 2017 |
| CA 121 | **A** | 100 | *F. columnare* | Eagle Lake trout (*Oncorhynchus mykiss aquilarum)* | CA | 2017 |
| CA 124 | **A** | 100 | *F. columnare* | Coho salmon (*O.* *kisutch*) | CA | 2017 |
| CA 126 | **A** | 100 | *F. columnare* | Coho salmon (*O.* *kisutch*) | CA | 2017 |
| CA 130 | **A** | 100 | *F. columnare* | Coho salmon (*O.* *kisutch*) | CA | 2017 |
| CA 131 | **A** | 100 | *F. columnare* | Coho salmon (*O.* *kisutch*) | CA | 2017 |
| CA 136 | **A** | 100 | *F. columnare* | Koi (*Cyprinos rubrofuscus*) | CA | 2018 |
| CA 137 | **A** | 100 | *F. columnare* | Koi (*C. rubrofuscus*) | CA | 2018 |
| CA 175 | **A** | 100 | *F. columnare* | Unknown fish | CA | 2018 |
| CA 186 | **A** | 100 | *F. columnare* | Rainbow trout (*Oncorhynchus mykiss*) | CA | 2018 |
| CA 193 | **A** | 100 | *F. columnare* | Steelhead (*O. mykiss*) | CA | 2018 |
| ID 1 | **A** | 100 | *F. columnare* | Rainbow trout (*O. mykiss*) | ID | 2021 |
| WA 166 | **A** | 100 | *F. columnare* | Coho salmon (*O.* *kisutch*) | WA | 2015 |
| WA 167 | **A** | 100 | *F. columnare* | Coho salmon (*O.* *kisutch*) | WA | 1991 |
| WA 168 | **A** | 100 | *F. columnare* | Chinook salmon (*Oncorhynchus tshawytscha*) | WA | 2014 |
| CA 123 | **A** | 99.9 | *F. covae* | Zebrafish (*Danio rerio*) | CA | 2017 |
| CA 59 | **A** | 99.9 | *F. inkyongense* | Chocolate cichlid (*Hypselecara temporalis*) | CA | 2017 |
| CA 211 | **A** | 99.7 | *F. davisii* | Rainbow trout (*O. mykiss*) | CA | 2018 |
| **CA 16** | **B** | 98.7 | *F. cupreum* | Kokanee salmon (*Oncorhynchus nerka*) | CA | 2015 |
| **CA 21** | **B** | 98.0 | *F. hibernum* | Red band trout (*Oncorhynchus mykiss stonei*) | CA | 2016 |
| CA 155 | **B** | 98.4 | *F. branchiarum* | Coho salmon (*O. kisutch*) | CA | 2018 |
| **UT 3gHT** | **B** | 98.4 | *F. branchiarum* | Rainbow trout (*O. mykiss*) | UT | 2021 |
| UT 4DT | **B** | 98.4 | *F. branchiarum* | Rainbow trout (*O. mykiss*) | UT | 2021 |
| CA 176 | **C** | 100 | *F. muglaense* | Steelhead (*O. mykiss*) | CA | 2018 |
| CA 177 | **C** | 100 | *F. muglaense* | Steelhead (*O. mykiss*) | CA | 2018 |
| **CA 178** | **C** | 100 | *F. muglaense* | Steelhead (*O. mykiss*) | CA | 2018 |
| **CA 66** | **C** | 99.9 | *F. muglaense* | Coho salmon (*O. kisutch*) | CA | 1995 |
| **UT 1AT** | **C** | 99.9 | *F. muglaense* | Rainbow trout (*O. mykiss*) | UT | 2020 |
| **CA 24** | **D** | 99.9 | *F. succinicans* | Eagle Lake trout (*O. mykiss aquilarum)* | CA | 2016 |
| **CA R2146 A** | **D** | 99.9 | *F. succinicans* | Tilapia (*Oreochromis* sp.) | CA | 2021 |
| **ID 10** | **D** | 99.1 | *F. succinicans* | Rainbow trout (*O. mykiss*) | ID | 2021 |
| ID 13 | **D** | 98.9 | *F. succinicans* | Rainbow trout (*O. mykiss*) | ID | 2021 |
| OR 38 | **D** | 99 | *F. succinicans* | Unknown fish | OR | Unknown |
| CA 116 | **D** | 98.2 | *F. tiangeerense* | Coho salmon (*O. kisutch*) | CA | 2017 |
| CA 118 | **D** | 98.2 | *F. tiangeerense* | Coho salmon (*O. kisutch*) | CA | 2017 |
| CA 220 | **D** | 98.2 | *F. tiangeerense* | White sturgeon (*Ascipenser transmontanus*) | CA | 2018 |
| **CA 61** | **E** | 98 | *F. bizetiae/commune/tiangeerense* | Rainbow trout (*O. mykiss*) | CA | 2017 |
| CA 65 | **E** | 98 | *F. bizetiae/commune/tiangeerense* | Chinook salmon (*O. tshawytscha*) | CA | 1994 |
| CA 68 | **E** | 98 | *F. bizetiae/commune/tiangeerense* | Chinook salmon (*O. tshawytscha*) | CA | 1995 |
| **Isolate name** | **Clade** | **Pairwise ID (%)** | **Type strain species** | **Host** | **State** | **Date** |
| CA 23 | **E** | 97.9 | *F. commune/tiangeerense* | Unknown | CA | 2016 |
| **CA 30** | **E** | 97.9 | *F. commune/tiangeerense* | Rainbow trout (*O. mykiss*) | CA | 2016 |
| CA 29 | **E** | 97.5 | *F. frigidimaris/glycines/micromati* | Steelhead (*O. mykiss*) | CA | 2016 |
| **CO 4SC** | **E** | 97.8 | *F. taihuense* | Brown trout (*Salmo trutta*) | CO | 2020 |
| OR 1-21 | **F** | 100 | *F. psychrophilum* | Coho salmon (*O. kisutch*) | OR | 2021 |
| OR 9-19 | **F** | 100 | *F. psychrophilum* | Chinook salmon (*O. tshawytscha*) | OR | 2022 |
| CA 156 | **F** | 99.2 | *F. psychrophilum* | Rainbow trout (*O. mykiss*) | CA | 2018 |
| CA 158 | **F** | 99.2 | *F. psychrophilum* | Rainbow trout (*O. mykiss*) | CA | 2018 |
| CA 169 | **F** | 99.2 | *F. psychrophilum* | Rainbow trout (*O. mykiss*) | CA | 2018 |
| CA 22 | **F** | 99.2 | *F. psychrophilum* | Rainbow trout (*O. mykiss*) | CA | 2016 |
| CA 28 | **F** | 99.2 | *F. psychrophilum* | Rainbow trout (*O. mykiss*) | CA | 2016 |
| CA 62 | **F** | 99.2 | *F. psychrophilum* | Rainbow trout (*O. mykiss*) | CA | 2017 |
| CA 63 | **F** | 99.2 | *F. psychrophilum* | Brown trout (*S. trutta*) | CA | 2017 |
| OR 11-19 | **F** | 99.2 | *F. psychrophilum* | Rainbow trout (*O. mykiss*) | OR | 2022 |
| OR 2-19 | **F** | 99.2 | *F. psychrophilum* | Chinook salmon (*O. tshawytscha*) | OR | 2019 |
| OR 4-19 | **F** | 99.2 | *F. psychrophilum* | Steelhead (*O. mykiss)* | OR | 2019 |
| OR 5-21 | **F** | 99.2 | *F. psychrophilum* | Chinook salmon (*O. tshawytscha*) | OR | 2021 |
| OR 6-21 | **F** | 99.2 | *F. psychrophilum* | Chinook salmon (*O. tshawytscha*) | OR | 2021 |
| OR 7-19 | **F** | 99.2 | *F. psychrophilum* | Chinook salmon (*O. tshawytscha*) | OR | 2022 |
| OR 8-19 | **F** | 99.2 | *F. psychrophilum* | Chinook salmon (*O. tshawytscha*) | OR | 2022 |
| CA 128 | **G** | 100 | *F. oncorhynchi* | Coho salmon (*O.* *kisutch*) | CA | 2017 |
| **CA 135** | **G** | 100 | *F. oncorhynchi* | Koi (*C. rubrofuscus*) | CA | 2018 |
| CA 147 | **G** | 100 | *F. oncorhynchi* | Koi (*C. rubrofuscus*) | CA | 2018 |
| CA 103 | **G** | 100 | *F. plurextorum* | Steelhead (*O. mykiss*) | CA | 2017 |
| **UT 39agC** | **G** | 100 | *F. plurextorum* | Rainbow trout (*O. mykiss*) | UT | 2020 |
| UT 39bgCT | **G** | 100 | *F. plurextorum* | Rainbow trout (*O. mykiss*) | UT | 2020 |
| WA 9563 | **G** | 100 | *F. plurextorum* | Steelhead (*O. mykiss*) | WA | 2014 |
| WA 9564 | **G** | 100 | *F. plurextorum* | Steelhead (*O. mykiss*) | WA | 2014 |
| **WA UI-A1** | **G** | 100 | *F. plurextorum* | Rainbow trout (*O. mykiss*) | WA | 2020 |
| WA UI-B1 | **G** | 99.9 | *F. plurextorum* | Rainbow trout (*O. mykiss*) | WA | 2020 |
| CA 76 | **H** | 99.1 | *F. salmonis* | Lahontan cutthroat trout (*Oncorhynchus clarkii* *henshawi)* | CA | 2017 |
| CA 110 | **H** | 98.4 | *F. salmonis* | Eagle Lake trout (*O. mykiss aquilarum)* | CA | 2017 |
| **CA 203** | **H** | 98.4 | *F. salmonis* | Rainbow trout (*O. mykiss*) | CA | 2018 |
| CA 215 | **H** | 99.1 | *F. salmonis* | Eagle Lake trout (*O. mykiss aquilarum)* | CA | 2018 |
| CA 112 | **H** | 99.3 | *F. tructae* | Rainbow trout (*O. mykiss*) | CA | 2017 |
| CA 133 | **H** | 99.6 | *F. tructae* | *Gambusia* sp. | CA | 2017 |
| CA 185 | **H** | 99.1 | *F. tructae* | Lahontan cutthroat trout (*O. clarkii* *henshawi)* | CA | 2018 |
| CA 218 | **H** | 100 | *F. tructae* | Steelhead (*O. mykiss*) | CA | 2018 |
| CA 70 | **H** | 99.2 | *F. tructae* | Coho salmon (*O.* *kisutch*) | CA | 2017 |
| CA 75 | **H** | 99.6 | *F. tructae* | Lahontan cutthroat trout (*O. clarkii* *henshawi)* | CA | 2017 |
| CA 97 | **H** | 99.6 | *F. tructae* | Rainbow trout (*O. mykiss*) | CA | 2017 |
| **CO 2S** | **H** | 99.2 | *F. tructae* | Brown trout (S. *trutta*) | CO | 2020 |
| CO 4SA | **H** | 99.2 | *F. tructae* | Brown trout (*S. trutta*) | CO | 2020 |
| CO 4SB | **H** | 99.2 | *F. tructae* | Brown trout (*S. trutta*) | CO | 2020 |
| **WA MC-02-10** | **H** | 98.4 | *F. tructae* | Rainbow trout (*O. mykiss*) | WA | 2020 |
| ID 4 | **H** | 98 | *F. chungangense* | Rainbow trout (*O. mykiss*) | ID | 2021 |
| WA 13688 | **H** | 98 | *F. chungangense* | Chinook salmon (*O. tshawytscha*) | WA | 2018 |
| WA 3497 | **H** | 98 | *F. chungangense* | Unknown | WA | 2008 |
| **Isolate name** | **Clade** | **Pairwise ID (%)** | **Type strain species** | **Host** | **State** | **Date** |
| CA 142 | **I** | 98.8 | *F. salmonis* | Rainbow trout (*O. mykiss*) | CA | 2018 |
| **CA 143** | **I** | 98.8 | *F. salmonis* | Rainbow trout (*O. mykiss*) | CA | 2018 |
| CA 210 | **I** | 98.8 | *F. salmonis* | Rainbow trout (*O. mykiss*) | CA | 2018 |
| CA 102 | **I** | 99.9 | *F. collinsii* | Steelhead (*O. mykiss*) | CA | 2017 |
| CA 12 | **I** | 99.9 | *F. collinsii* | Steelhead (*O. mykiss*) | CA | Unknown |
| CA 148 | **I** | 99.7 | *F. collinsii* | Chinook salmon (*O. tshawytscha*) | CA | 2018 |
| CA 201 | **I** | 99.7 | *F. collinsii* | Rainbow trout (*O. mykiss*) | CA | 2018 |
| CA 202 | **I** | 99.7 | *F. collinsii* | Rainbow trout (*O. mykiss*) | CA | 2018 |
| CA 219 | **I** | 99.7 | *F. collinsii* | White sturgeon (*A. transmontanus*) | CA | 2018 |
| CA 221 | **I** | 99.7 | *F. collinsii* | White sturgeon (*A. transmontanus*) | CA | 2018 |
| CA 25 | **I** | 99.9 | *F. collinsii* | Coho salmon (*O. kisutch*) | CA | 2016 |
| CA 94 | **I** | 99.9 | *F. collinsii* | Chinook salmon (*O. tshawytscha*) | CA | 2017 |
| ID 6 | **I** | 99.7 | *F. collinsii* | Rainbow trout (*O. mykiss*) | ID | 2022 |
| **ID 7** | **I** | 99.7 | *F. collinsii* | Rainbow trout (*O. mykiss*) | ID | 2022 |
| ID 8 | **I** | 99.9 | *F. collinsii* | Rainbow trout (*O. mykiss*) | ID | 2022 |
| OR 48 | **I** | 100 | *F. collinsii* | Unknown | OR | 1996 |
| **WA 11299** | **I** | 99.7 | *F. collinsii* | Rainbow trout (*O. mykiss*) | WA | 2018 |
| WA 13146 | **I** | 99.7 | *F. collinsii* | Rainbow trout (*O. mykiss*) | WA | 2018 |
| CA 20 | N/A | 98.8 | *F. taihuense* | Coho salmon (*O. kisutch*) | CA | 2016 |
| CA 104 | N/A | 97.9 | *F. taihuense* | Steelhead (*O. mykiss*) | CA | 2017 |
| CO 4SD | N/A | 97.8 | *F. taihuense* | Brown trout (*S. trutta*) | CO | 2020 |
| OR 39 | N/A | 97.6 | *F. taihuense* | Unknown | OR | Unknown |
| OR 2 | N/A | 100 | *F. bizetiae* | Steelhead (*O. mykiss*) | OR | Unknown |
| CA R1970 3 | N/A | 99.1 | *F. bizetiae* | Chinook salmon (*O. tshawytscha*) | CA | Unknown |
| CA R1985 20 | N/A | 98.4 | *F. bizetiae* | Chinook salmon (*O. tshawytscha*) | CA | Unknown |
| WA 13472 | N/A | 98.4 | *F. bizetiae* | Chinook salmon (*O. tshawytscha*) | WA | 2019 |
| OR 10-19 | N/A | 99.6 | *F. erciyesense* | Rainbow trout (*O. mykiss*) | OR | 2022 |
| CA 8 | N/A | 99.3 | *F. erciyesense* | Eagle Lake trout (*O. mykiss aquilarum*) | CA | 2015 |
| UT 19-058 | N/A | 98.4 | *F. granuli* | Rainbow trout (*O. mykiss*) | UT | 2019 |
| UT 2lesAT | N/A | 98.3 | *F. granuli* | Rainbow trout (*O. mykiss*) | UT | 2021 |
| CA 31 | N/A | 98.4 | *F. branchiicola/oncorhynchi* | Unknown | CA | Unknown |
| UT 4gAT | N/A | 98.4 | *F. branchiicola/oncorhynchi* | Rainbow trout (*O. mykiss*) | UT | 2021 |
| UT 04-081 | N/A | 99.2 | *F. branchiicola* | Rainbow trout (*O. mykiss*) | UT | 2004 |
| WA 2319 | N/A | 98.9 | *F. aquidurense* | Unknown | WA | 2005 |
| UT 10gBT | N/A | 97.3 | *F. aquidurense* | Rainbow trout (*O. mykiss*) | UT | 2020 |
| CA 41 | N/A | 99.7 | *F. aquaticum* | Green sturgeon (*Ascipenser medirostris*) | CA | 2016 |
| CA R1985 5 | N/A | 99.7 | *F. phocarum* | Chinook salmon (*O. tshawytscha*) | CA | Unknown |
| WA MC-02-09 | N/A | 98.8 | *F. hercynium* | Rainbow trout (*O. mykiss*) | WA | 2020 |
| WA BC-01-14 | N/A | 98.7 | *F. cupreum* | Rainbow trout (*O. mykiss*) | WA | 2020 |
| CA 132 | N/A | 98.5 | *F. chungangense* | Rainbow trout (*O. mykiss*) | CA | 2017 |
| CA 139 | N/A | 98.1 | *F. terrigena* | Steelhead (*O. mykiss*) | CA | 2018 |
| CA R1985 6 | N/A | 97.2 | *F. banpakuense* | Chinook salmon (*O. tshawytscha*) | CA | Unknown |
| CA 217 | N/A | 97.9 | *F. sinopsychrotolerans* | Steelhead (*O. mykiss*) | CA | 2018 |
| CA 68B | N/A | 95.8 | *F. supellecticarium* | Goldfish (C*arassius auratus*) | CA | 2021 |

**STable 4:** Isolates collected from diseased fish in the Western US identified as *Chryseobacterium* species. The *gyrB* clade designation and coloration reflect genetic groupings in corresponding phylogenetic trees (Fig. 2). The closest accepted species was determined by BLAST alignment of isolate 16S rRNA sequence fragments to a local database populated by type-strain 16S rRNA genes. Pairwise ID is the average percent identity over the alignment. Isolates in bold were used in antimicrobial testing.

| **Isolate name** | **Clade** | **Pairwise ID (%)** | **Type strain species** | **Host** | | **State** | **Date** |
| --- | --- | --- | --- | --- | --- | --- | --- |
| CA 107 | **A** | 99.5 | *C. chaponense* | Steelhead (*O. mykiss*) | CA | | 2017 |
| CA 114 | **A** | 99.5 | *C. chaponense* | Rainbow trout (*O. mykiss*) | CA | | 2017 |
| **CA 144** | **A** | 99.5 | *C. chaponense* | Rainbow trout (*O. mykiss*) | CA | | 2018 |
| CA 145 | **A** | 99.5 | *C. chaponense* | Rainbow trout (*O.* *mykiss*) | CA | | 2018 |
| **CA 205** | **A** | 99.5 | *C. chaponense* | Rainbow trout (*Oncorhynchus mykiss*) | CA | | 2018 |
| CA 222 | **A** | 99.5 | *C. chaponense* | Rainbow trout (*O. mykiss*) | CA | | 2021 |
| **WA SH-05-01** | **A** | 99.5 | *C. chaponense* | Rainbow trout (*O. mykiss*) | WA | | 2020 |
| **WA MC-02-13** | **B** | 100 | *C. piscicola* | Rainbow trout (*O. mykiss*) | WA | | 2020 |
| UT 0412BT | **B** | 99.9 | *C. piscicola* | Rainbow trout (*O. mykiss*) | UT | | 2021 |
| CA 6 | **B** | 99.9 | *C. piscicola* | Rainbow trout (*O. mykiss*) | CA | | 2016 |
| CA R1970 5 | **B** | 99.6 | *C. piscicola* | Chinook salmon (*Oncorhynchus tshawytscha)* | CA | | Unk |
| ID 9 | **B** | 99.6 | *C. piscicola* | Rainbow trout (*O. mykiss*) | ID | | 2017 |
| **WA UI-1G** | **B** | 99.6 | *C. piscicola* | Rainbow trout (*O. mykiss*) | WA | | 2014 |
| **WA UI-3B** | **B** | 99.6 | *C. piscicola* | Rainbow trout (*O. mykiss*) | WA | | 2014 |
| WA UI-8G | **B** | 99.6 | *C. piscicola* | Rainbow trout (*O. mykiss*) | WA | | 2014 |
| WA NS-910-01 | **B** | 98.4 | *C. aquaticum* | Rainbow trout (*O. mykiss*) | WA | | 2019 |
| **WA 9757** | **C** | 99.5 | *C. schmidteae* | Rainbow trout (*O. mykiss*) | WA | | 2015 |
| **ID 2** | **C** | 99.4 | *C. scopthalmum* | Rainbow trout (*O. mykiss*) | ID | | 2019 |
| WA NS-96-11 | **C** | 99.3 | *C. mulctrae* | Rainbow trout (*O. mykiss*) | WA | | 2019 |
| CA R2137 | **C** | 98.4 | *C. mulctrae* | Koi (*C. rubrofuscus*) | CA | | 2021 |
| **CA 134** | **C** | 97.8 | *C. mulctrae* | Koi (*Cyprinus rubrofuscus*) | CA | | 2018 |
| **WA NS-95-19** | **D** | 99.8 | *C. carnis* | Rainbow trout (*O. mykiss*) | WA | | 2019 |
| **WA SH-05-02** | **D** | 99.5 | *C. chaponense* | Rainbow trout (*O. mykiss*) | WA | | 2020 |
| CA R1970 2 | **D** | 98.9 | *C. piscium* | Chinook salmon (*O. tshawytscha*) | CA | | Unk |
| CA R1970 4 | **D** | 98.9 | *C. piscium* | Chinook salmon (*O. tshawytscha*) | CA | | Unk |
| WA NS-05-07 | **D** | 98.9 | *C. piscium* | Rainbow trout (*O. mykiss*) | WA | | 2020 |
| **WA NS-05-04** | **D** | 97.2 | *C. salvictor* | Rainbow trout (*O. mykiss*) | WA | | 2020 |
| WA NS-96-16 | **D** | 97.2 | *C. salvictor* | Rainbow trout (*O. mykiss*) | WA | | 2019 |
| WA NS-96-18 | **D** | 97.2 | *C. salvictor* | Rainbow trout (*O. mykiss*) | WA | | 2019 |
| WA BC-05-03 | **E** | 100 | *C. oncorhynchi* | Rainbow trout (*O. mykiss*) | WA | | 2020 |
| **WA UI-4G** | **E** | 99.3 | *C. oncorhynchi* | Rainbow trout (*O. mykiss*) | WA | | 2014 |
| **WA UI-6B** | **E** | 99.3 | *C. oncorhynchi* | Rainbow trout (*O. mykiss*) | WA | | 2014 |
| CA R1970 11 | **E** | 99.9 | *C. ureilyticum* | Chinook salmon (*O. tshawytscha*) | CA | | Unk |
| **CA R2136** | **E** | 98.9 | *C. oncorhynchi* | Tilapia (*Oreochromis* sp.) | CA | | 2021 |
| CA 146 | **E** | 95.6 | *C. aurantiacum* | Koi (*C. rubrofuscus*) | CA | | 2018 |
| **Isolate name** | **Clade** | **Pairwise ID (%)** | **Type strain species** | **Host** | **State** | | **Date** |
| **CA 127** | **F** | 99.9 | *C. indologenes* | Coho salmon (*Oncorhynchus* *kisutch*) | CA | | 2017 |
| **CA 129** | **F** | 99.9 | *C. indologenes* | Coho salmon (*O. kisutch*) | CA | | 2017 |
| **CA 44** | **F** | 99.2 | *C. capnotolerans* | White sturgeon (*A. transmontanus*) | CA | | 2016 |
| CA 71 | **F** | 99.1 | *C. ureilyticum* | Blue ram cichlid (*Mikrogeophagus* *ramirezi*) | CA | | 2017 |
| CA 46 | **F** | 98.5 | *C. jejuense* | White sturgeon (*Acipenser transmontanus*) | CA | | 2016 |
| CA 43 | N/A | 100 | *C. aquaticum* | Green sturgeon (*Acipenser medirostris*) | CA | | 2016 |
| CA 213 | N/A | 100 | *C. gambrini* | Rainbow trout (*O. mykiss*) | CA | | 2018 |
| CA R2154 D | N/A | 99.7 | *C. candidae* | Koi (*C. rubrofuscus*) | CA | | 2021 |
| ID 11 | N/A | 99.7 | *C. vrystaatense* | Rainbow trout (*O. mykiss*) | ID | | 2017 |
| ID 12 | N/A | 99.7 | *C. vrystaatense* | Rainbow trout (*O. mykiss*) | ID | | 2017 |
| ID 3 | N/A | 99.6 | *C. lecithinasegans* | Rainbow trout (*O. mykiss*) | ID | | 2019 |
| CA 196 | N/A | 99.2 | *C. soli* | Steelhead (*O. mykiss*) | CA | | 2018 |
| UT SK1 | N/A | 99.2 | *C. antibioticum* | June sucker (*Chasmistes liorus*) | UT | | 2004 |
| CA 15 | N/A | 99.2 | *C. candidae* | Rainbow trout (*O. mykiss*) | CA | | 2016 |
| CA 68E | N/A | 98.8 | *C. mulctrae* | Goldfish (*Carassius auratus*) | CA | | 2021 |
| CA 161 | N/A | 98.4 | *C. gregarium* | Coho salmon (*O. kisutch*) | CA | | 2018 |
| CA 45 | N/A | 98.3 | *C. nakagawai* | White sturgeon (*A. transmontanus*) | CA | | 2016 |


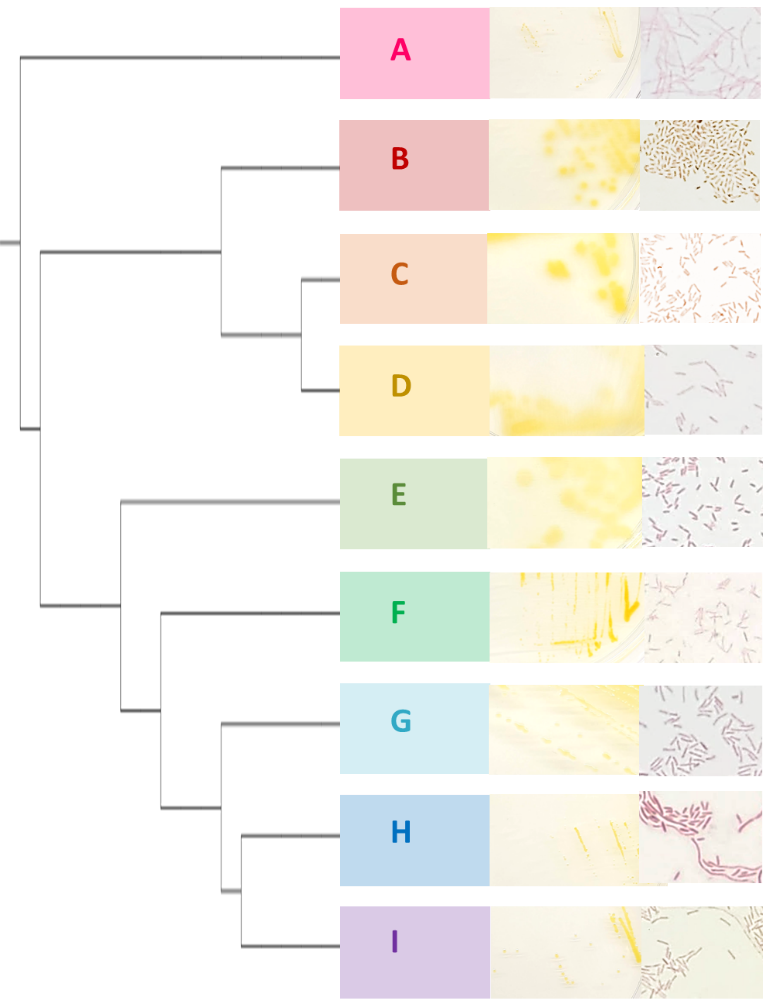


**SFigure 1:** Morphological characteristics of representative *Flavobacterium* isolates from each major *gyrB* phylogenetic clade. *Flavobacterium* isolates were gram-negative rods of varying length and thickness that formed yellow or translucent yellow round or spreading colonies on solid media. Clade A included established pathogen *F. columnare* and Clade F, *F.* *psychrophilum*.


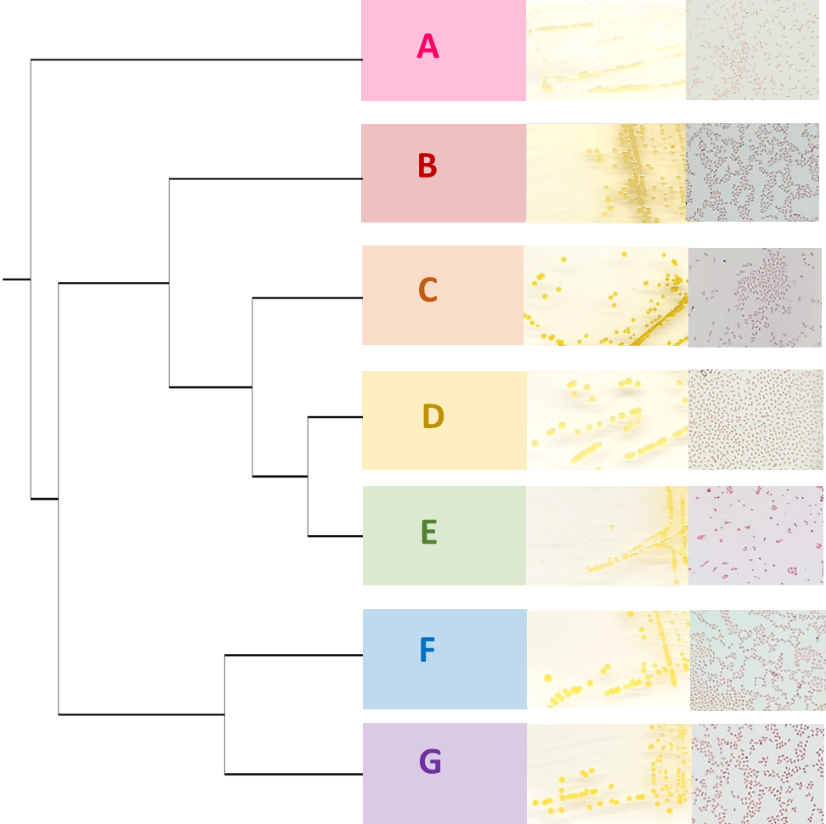


**SFigure 2:** Morphological characteristics of representative *Chryseobacterium* isolates from each major *gyrB* phylogenetic clade. Chryseobacterium isolates were short, rounded gram-negative rods that formed pigmented colonies ranging from translucent yellow, vibrant yellow, gray-yellow, and dark gold.


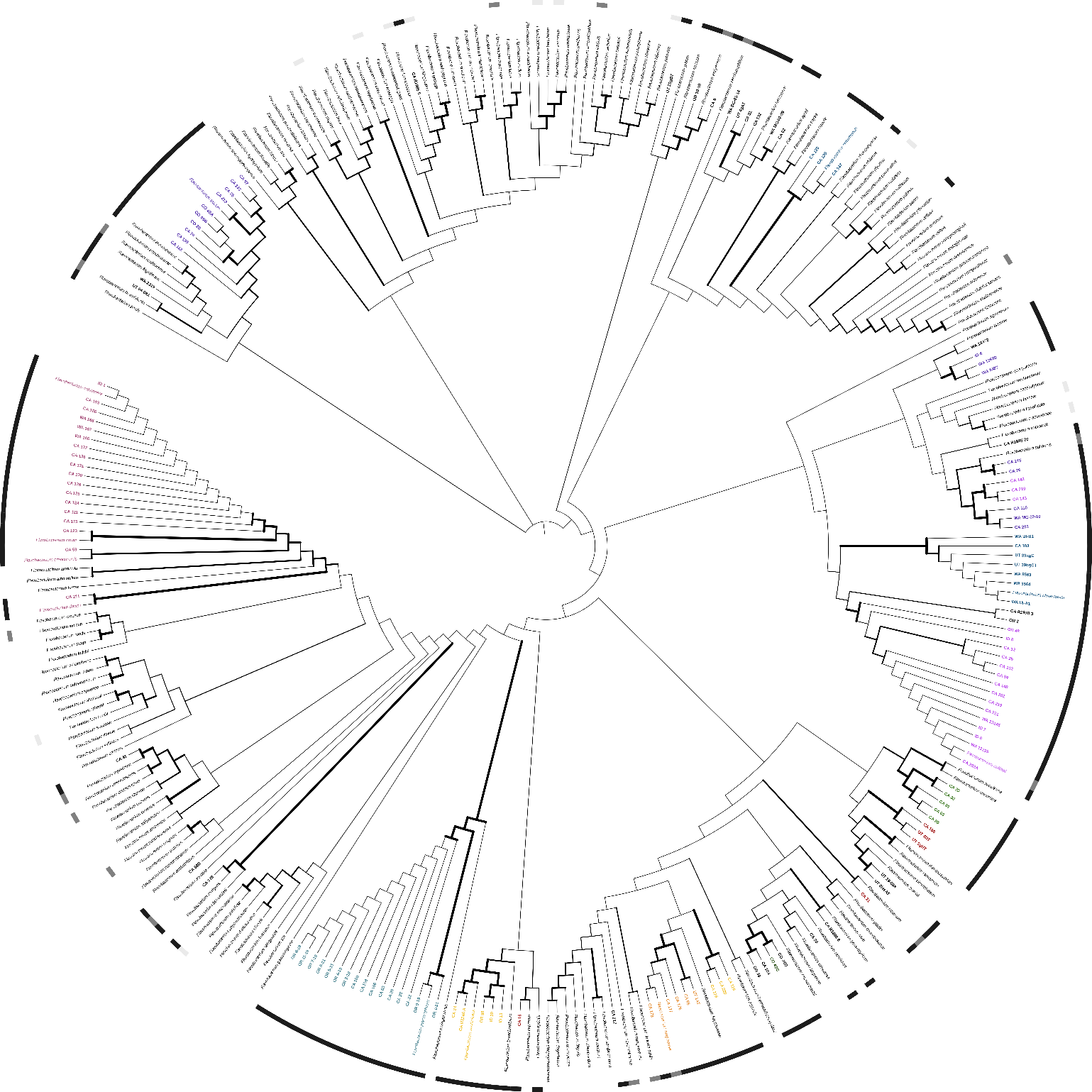


**SFigure 3**: Phylogenetic relationships of 131 *Flavobacterium* spp. isolates recovered from diseased fishes in the Western US to 178 formally described *Flavobacterium* spp., based on partial 16S rRNA gene sequences. The dendrogram was generated using the Maximum-Likelihood method and General Time Reversible model with a Gamma distribution allowing for evolutionarily invariable sites (GTR+G+I) and 1000 pseudoreplicates. Branch width is proportional to bootstrap support with increasing thickness from 0-1. Color strips indicate host relationship: black – fish disease associated, dark grey – fish associated, light gray – non-fish animal associated. Text color indicates clade in the corresponding *gyrB* tree (Figure 1): A – pink, B – red, C – orange, D – yellow, E – light green, F – teal, G – light blue, H – dark blue, I - purple. An interactive version of this tree can be accessed at <https://itol.embl.de/tree/31386026429741670886914>


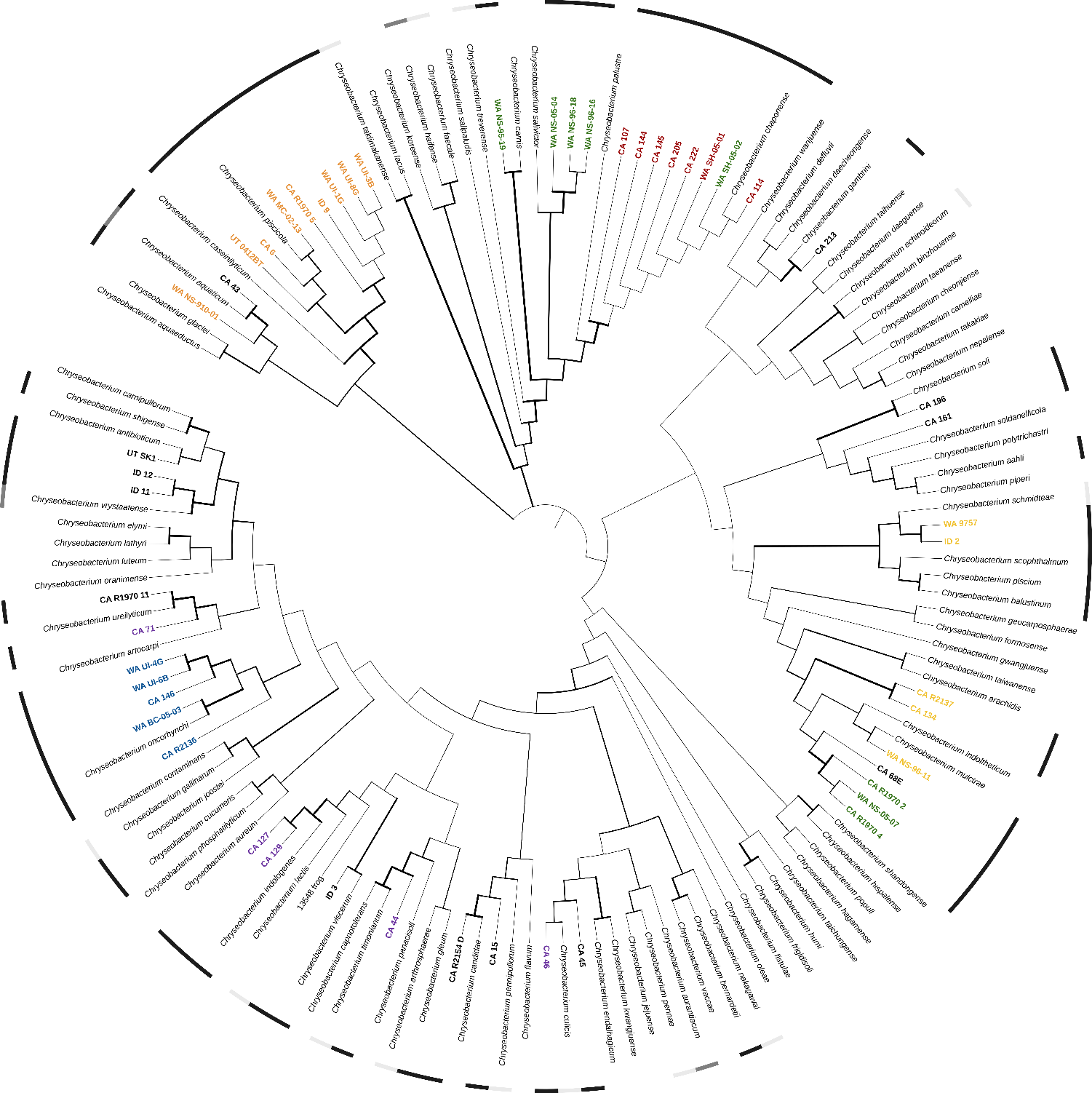


**SFigure 4:** Phylogenetic relationships of 53 *Chryseobacterium* spp. isolates recovered from diseased fishes in the Western US to 94 formally described *Chryseobacterium* spp., based on partial 16S rRNA gene sequences. The dendrogram was generated using the Maximum-Likelihood method and General Time Reversible model with a Gamma distribution allowing for evolutionarily invariable sites (GTR+G+I) and 1000 pseudoreplicates. Branch width is proportional to bootstrap support with increasing thickness from 0-1. Color strips indicate host relationship: black – fish disease associated, dark grey – fish associated, light gray – non-fish animal associated. Text color indicates clade in corresponding *gyrB* tree (Figure 3): A – pink, B – red, C – orange, D – yellow, E – green, F – blue, G – purple. An interactive version of this tree can be accessed at <https://itol.embl.de/tree/31386026427871670886843>
